# Mechanism of hERG inhibition by gating-modifier toxin, APETx1, deduced by functional characterization

**DOI:** 10.1186/s12860-020-00337-3

**Published:** 2021-01-07

**Authors:** Kazuki Matsumura, Takushi Shimomura, Yoshihiro Kubo, Takayuki Oka, Naohiro Kobayashi, Shunsuke Imai, Naomi Yanase, Madoka Akimoto, Masahiro Fukuda, Mariko Yokogawa, Kazuyoshi Ikeda, Jun-ichi Kurita, Yoshifumi Nishimura, Ichio Shimada, Masanori Osawa

**Affiliations:** 1 Graduate School of Pharmaceutical Sciences, Keio University, Shibakoen, Minato-ku, Tokyo, 105-8512 Japan; 2 Division of Biophysics and Neurobiology, Department of Molecular and Cellular Physiology, National Institute for Physiological Sciences, Myodaiji, Okazaki-shi, Aichi 444-8585 Japan; 3 Nanion Technologies Japan K.K., Tokyo Laboratory, Wakamatsu-cho, Shinjuku-ku, Tokyo, 162-0056 Japan; 4 Institute for Protein Research, Osaka University, Yamadaoka, Suita-shi, Osaka, 565-0871 Japan; 5 NMR Science and Development Division, RSC, RIKEN, Suehiro-cho, Tsurumi-ku, Yokohama-shi, Kanagawa 230-0045 Japan; 6 Graduate School of Pharmaceutical Sciences, The University of Tokyo, Hongo, Bunkyo-ku, Tokyo, 113-0033 Japan; 7 Graduate School of Medical Life Science, Yokohama City University, Suehiro-cho, Tsurumi-ku, Yokohama, Kanagawa 230-0045 Japan

## Abstract

**Background:**

Human *ether-à-go-go*-related gene potassium channel 1 (hERG) is a voltage-gated potassium channel, the voltage-sensing domain (VSD) of which is targeted by a gating-modifier toxin, APETx1. APETx1 is a 42-residue peptide toxin of sea anemone *Anthopleura elegantissima* and inhibits hERG by stabilizing the resting state. A previous study that conducted cysteine-scanning analysis of hERG identified two residues in the S3-S4 region of the VSD that play important roles in hERG inhibition by APETx1. However, mutational analysis of APETx1 could not be conducted as only natural resources have been available until now. Therefore, it remains unclear where and how APETx1 interacts with the VSD in the resting state.

**Results:**

We established a method for preparing recombinant APETx1 and determined the NMR structure of the recombinant APETx1, which is structurally equivalent to the natural product. Electrophysiological analyses using wild type and mutants of APETx1 and hERG revealed that their hydrophobic residues, F15, Y32, F33, and L34, in APETx1, and F508 and I521 in hERG, in addition to a previously reported acidic hERG residue, E518, play key roles in the inhibition of hERG by APETx1. Our hypothetical docking models of the APETx1-VSD complex satisfied the results of mutational analysis.

**Conclusions:**

The present study identified the key residues of APETx1 and hERG that are involved in hERG inhibition by APETx1. These results would help advance understanding of the inhibitory mechanism of APETx1, which could provide a structural basis for designing novel ligands targeting the VSDs of K_V_ channels.

## Background

Human *ether-à-go-go*-related gene potassium channel 1 (hERG; K_V_11.1) is a voltage-gated potassium channel (K_V_ channel) expressed in human cardiomyocytes, as well as brain and cancer cells [1–3]. hERG conducts potassium ions (K^+^) across the cell membrane upon depolarization, thereby contributing to the repolarization of the action potential [2, 3]. This function is necessary for a normal heartbeat, as demonstrated by the fact that some hERG inhibitors cause lethal arrhythmia accompanied by long QT syndrome [2, 4–6]. Recently, it has been reported that variations in the gene encoding hERG are associated with schizophrenia [7–9], and that alterations in hERG expression and function are observed in various types of cancer cells and are involved in carcinogenic processes [10–12]. Non-arrhythmogenic hERG inhibitors, which block hERG without inducing arrhythmia, can improve the survival rate among glioblastoma patients showing high hERG expression [13]. These clinical results demonstrate the therapeutic potential of the specific ligands controlling hERG function; thus, it is of great medical importance to determine the structural mechanisms underlying the interactions between hERG and its specific ligands [3, 12, 14].

hERG is a tetrameric channel in which each subunit comprises six transmembrane segments (S1-S6) and N- and C-terminal cytoplasmic domains [1, 15–18]. In the tetrameric architecture of K_V_ channels, the S5 and S6 segments form a pore domain (PD) with a K^+^-selective filter and an activation gate at the center of the tetramer, and the S1-S4 segments of each subunit form a voltage-sensing domain (VSD) at the four peripheries of the PD [17, 19–23]. The voltage-dependent conformational changes of the VSD regulate the opening and closing of the gate in the PD [24–26]. Upon membrane depolarization, the VSD undergoes a conformational change from “S4-down” to “S4-up,” in which S4 moves from the intracellular side to the extracellular side roughly perpendicular to the membrane plane [24–26]. To date, three-dimensional hERG structures have been determined by cryo-electron microscopy (cryo-EM), which has revealed that the VSD adopts the S4-up conformation under the nominal absence of membrane potential in detergent micelles [17]. By contrast, the resting-state structure, in which the VSD adopts the S4-down conformation, has not been determined because structural analysis under resting membrane potential is technically challenging.

Specific ligands that stabilize the resting state of hERG include APETx1, which is a 42-amino-acid peptide toxin of the sea anemone *Anthopleura elegantissima* [27]. APETx1 is a gating-modifier toxin that binds to the VSD and inhibits the voltage-dependent activation of hERG, thus stabilizing the resting-state, S4-down conformation of the VSD [27, 28]. Therefore, APETx1 is an effective tool for characterizing the molecular surface of the hERG VSD in the S4-down conformation, and for exploring the binding sites of the specific ligands that control hERG function.

A previous study that conducted cysteine-scanning analysis of hERG identified two residues in the S3-S4 region of the VSD that play important roles in hERG inhibition by APETx1 [28]. However, only natural resources have been available until now, and thus, mutational analysis of APETx1 could not be conducted. Therefore, no information could be obtained regarding the APETx1 residues crucial for hERG inhibition, and it remains unclear how APETx1 binds to the S4-down conformation of the VSD to inhibit hERG activation.

In the present study, we established a method for preparing recombinant APETx1 and investigated the hERG inhibition activity of APETx1 through electrophysiological analyses of APETx1 and hERG mutants. We identified the hydrophobic residues of APETx1, as well as those in the hERG S3-S4 region related to hERG inhibition by APETx1. Next, we constructed hypothetical docking models of APETx1-VSD complex that satisfy the results of mutational analysis. These results would help advance understanding of the inhibitory mechanism of hERG by APETx1.

## Results

### Functional and structural characterization of recombinant APETx1

In a previous study, APETx1 was purified from the sea anemone *Anthopleura elegantissima* (hereafter referred to as “natural product”) to characterize its inhibitory effect on hERG and its solution structure [27–29]. Here, we prepared recombinant APETx1, which was expressed as inclusion bodies in *E. coli*, purified in urea buffer, and refolded by dialysis. This dialysis process allowed the formation of three intramolecular disulfide bonds (see Materials and Methods for details of the sample preparation). Purified recombinant APETx1 is confirmed to show a single peak by reverse-phase high-performance liquid chromatography (RP-HPLC; Fig. S1a). The measured molecular weight of the natural product (4551.99 Da) was consistent with the molecular weight calculated using the sequence with three disulfide bonds (4552.21 Da), supporting the data indicating that there are no post-translational modifications in the natural product [27]. In the present study, the recombinant APETx1 showed a similar molecular weight (4551.267 Da; Fig. S1b), demonstrating chemical equivalence with the natural product. Therefore, we examined whether the prepared recombinant APETx1 is also equivalent to the natural product in terms of structure and function.

First, a set of amide proton chemical shifts of the recombinant APETx1 was compared with the corresponding set from the natural product at pH 3.0 and 280 K [29] in order to examine their structural equivalence. We established the NMR (nuclear magnetic resonance) assignments of the recombinant APETx1 at pH 6.0 and 298 K (Fig. S2a-c). Then, the assignments of the recombinant APETx1 at pH 3.0 and 280 K were obtained by performing pH-titration and variable-temperature measurements (Fig. S3a-d). It should be noted that the backbone amide signals of Y5, F33, and L34 were not observed in the ^1^H-^15^N HSQC (heteronuclear single quantum correlation) spectrum at pH 3.0 and 280 K. The chemical shift values of backbone amide protons uniformly deviated by 0.12 ppm on average from those previously reported (Fig. S3e) [29]. These differences are probably due to systematic errors, such as from chemical shift referencing. Taking into consideration these systematic differences, the chemical shift differences are within ±0.05 ppm, indicating that the structure of the recombinant APETx1 is identical to that of the natural product.

**Fig. 1:**
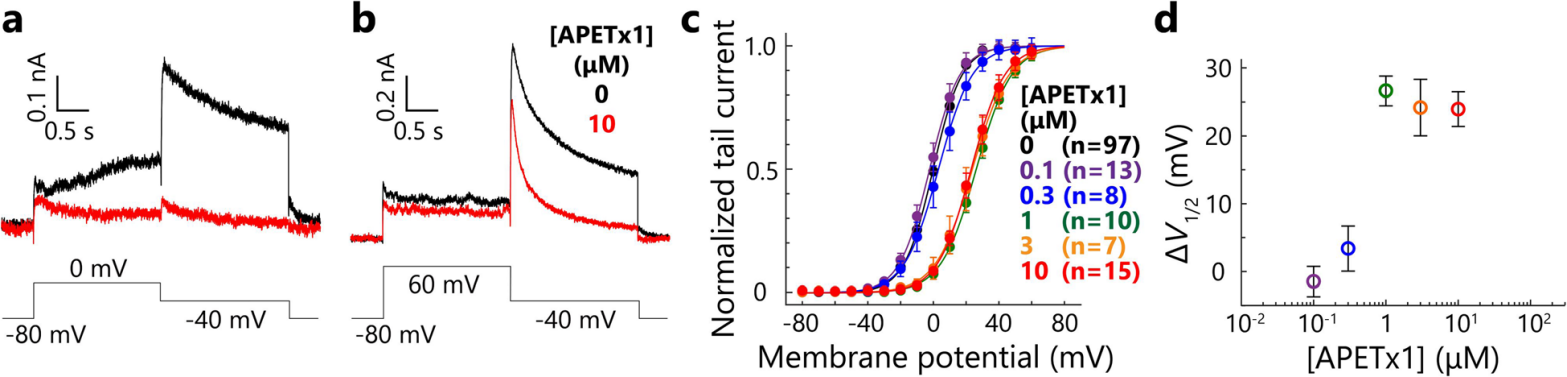
Functional and structural characterization of recombinant APETx1 in patch-clamp recordings. **a**, **b** Current traces of hERG elicited by two different depolarization pulses, 0 mV (**a**) and 60 mV (**b**), before (black) and after (red) the administration of 10 μM APETx1. Voltage protocol is illustrated at the bottom of each current trace. **c** Normalized *G*-*V* curves (mean ± SEM) of hERG in the presence or absence of different APETx1 concentrations. **d** The Δ*V*_1/2_ values of different concentrations of APETx1; these values indicate the toxin-induced shift of the half-maximal activation voltage of hERG

Next, the hERG inhibition effect of the recombinant APETx1 was evaluated and compared with that of the natural product [27, 28]. We observed hERG K^+^ currents in the presence or absence of the recombinant APETx1 by whole-cell patch-clamp recordings and two-electrode voltage clamp (TEVC) recordings. The data obtained from whole-cell patch-clamp recordings (Fig. 1a-d) demonstrated that the recombinant APETx1 effectively inhibited the hERG currents and shifted the half-maximal activation voltage (*V*_1/2_) values toward positive voltage in a dose-dependent manner. These results are consistent with those of previous studies [27, 28]. Similar results were obtained from TEVC recordings (Fig. S4a and c-d).

The apparent dissociation constant (*K*_d_) values for the binding of APETx1 to hERG were estimated based on the data obtained from TEVC recordings, according to a previously reported method [28]. The fraction of uninhibited currents (*I*_Toxin_ / *I*_Control_), which was derived from the tail currents at weak depolarizing pulses (− 30 mV) was reduced by incremental increases of APETx1, and the data were fitted with three models, (A)-(C), as described previously (Fig. S4b) [28]. Because residual uninhibited currents were observed even at the 10 μM concentration of APETx1, models (A) and (C), which assume fractional toxin-sensitive currents, fitted the data more closely than model (B), which assumes a fully toxin-sensitive current; this is consistent with the findings of a previous study [28]. It is presently unclear which model supports APETx1 binding to hERG. It should be noted that the calculated *K*_d_ values of the three models are as follows: (A) 1.2 μM, (B) 1.7 μM, and (C) 0.23 μM; these are 12- to 14-fold higher than *K*_d_ values from a previous study, which are: (A) 87 nM, (B) 141 nM, and (C) 16.3 nM [28]. Differences in the *K*_d_ values between the previous and present studies are unclear as the natural product was not available to us, making it difficult to conduct direct comparisons between the recombinant APETx1 and the natural product in identical experimental conditions.

### Solution structure of APETx1 at pH 6.0

**Fig. 2:**
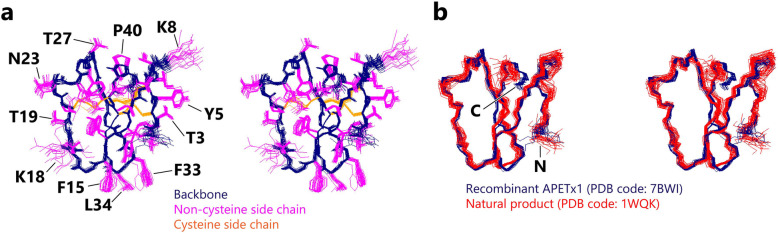
Structure determination of recombinant APETx1 at pH 6.0 and 298 K. **a** Stereo view of the ensemble of the 20 lowest-energy structures of recombinant APETx1, colored as follows: heavy atoms of backbone, navy; non-cysteine side chain, magenta; cysteine side chain, orange. **b** Stereo view of the overlay of the 20 structures of recombinant APETx1 at pH 6.0 and 298 K from the present study (PDB code: 7BWI; navy) and the 25 structures of the natural product at pH 3.0 and 280 K from a previous study (PDB code: 1WQK; red) [29]

We observed pH-dependent chemical shift changes in the backbone-amide signals between pH 3.0 and pH 6.0 for the C-terminal residues V41 and D42 and the side-chain signal of R24 (Fig. S3f). These results suggest that the previously reported structure, determined at pH 3.0 [29], might be different from that which occurs under physiological pH conditions. We therefore determined the three-dimensional structure of the recombinant APETx1 at pH 6.0 (Fig. 2a and Table 1), and compared this with the structure at pH 3.0 (Fig. 2b) [29]. Backbone overlay of these two structures clearly shows that the structure of the C-terminal region is different (Fig. 2b). At pH 6.0, the side-chain carboxyl group of D42 lies closer to the guanidinium group of R24, possibly because the deprotonation of the former enables the formation of hydrogen bonds or electrostatic interaction with the latter. pH-titration experiments showed that the changes in chemical shift values are reversible (data not shown). These results indicate that although the conformation of the C-terminal region is slightly altered according to pH conditions, the overall structure of APETx1 remains essentially identical.

**Table 1:** Structural statistics of the APETx1 structures in the present study (recombinant protein; 20 conformers) and previous study (natural product; 25 conformers) [27]

	Natural product [27]	Recombinant protein
PDB code	1WQK	7BWI
Experimental conditions	pH 3.0 and 280 K	pH 6.0 and 298 K
Distance restraints		
Total NOE-derived restraints	751	766
Intraresidue restraints (|i-j| = 0)	366	131
Sequential restraints (|i-j| = 1)	140	216
Short-range restraints (2 ≤ |i-j| ≤ 4)	61	94
Long-range restraints (|i-j| ≥ 5)	184	325
Disulfide bond restraints	9	12
Dihedral angle restraints	20	41
Hydrogen-bond restraints	36	–
Root-mean-square deviation (RMSD) from mean coordinate structure (Å)^a^		
Backbone heavy atoms		
Residues 1–42	0.82 ± 0.17	0.66 ± 0.21
Residues 2–41	0.63 ± 0.13	0.48 ± 0.12
All heavy atoms		
Residues 1–42	1.28 ± 0.17	1.01 ± 0.15
Residues 2–41	1.13 ± 0.15	0.95 ± 0.14
Analysis of the Ramachandran plot (%)^b^		
Residues in favored regions	84.3	93.7
Residues in allowed regions	14.0	5.6
Ramachandran outliers	1.7	0.6

^a^Root-mean-square deviation (RMSD) is calculated by MOLMOL [30]. ^b^Stereochemical quality is evaluated according to MolProbity (http://molprobity.biochem.duke.edu/)

### Four clustered hydrophobic residues of APETx1 contributing to hERG inhibition identified by mutational analysis

**Fig. 3:**
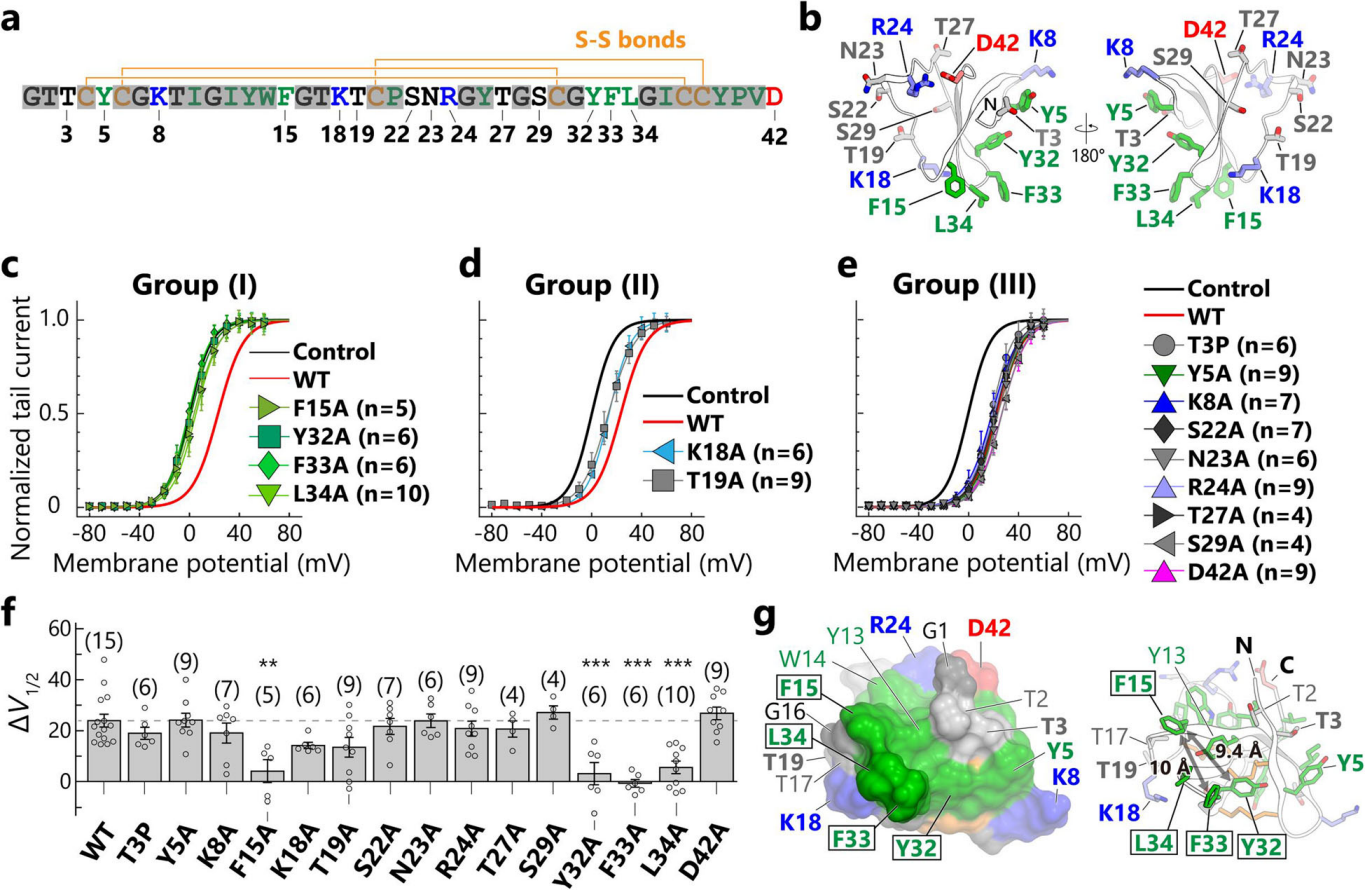
hERG inhibition by APETx1 mutants. **a** The primary structure of APETx1, colored as follows: positively-charged residues (K and R), blue; negatively-charged residues (D), red; hydrophobic residues (F, I, L, P, V, W, and Y), green; cysteine residues, orange; and others (G, N, S, and T), black. Three pairs of disulfide bonds are shown with orange lines. Non-mutated residues are drawn with semi-transparent gray marker to highlight the mutated residues. **b** The structure of APETx1 is shown as a ribbon representation with sticks depicting the mutated residues. **c**-**e** Normalized *G*-*V* curves (mean ± SEM) of hERG in the presence of 10 μM APETx1 mutants expressed by different colors and symbols. Control and WT correspond to the normalized *G*-*V* curve in the presence and absence, respectively, of 10 μM APETx1 from Fig. 1c. Based on the Δ*V*_1/2_ values, APETx1 mutants were categorized into the groups (I)-(III). **f** The Δ*V*_1/2_ values of 10 μM APETx1 mutants in hERG are represented as the mean values ± SEM, with the number of experiments shown in parentheses. Multiple-group comparison was performed by one-way ANOVA followed by Tukey’s test (∗, 0.01 ≤ *p* < 0.05; ∗∗, 0.001 ≤ *p* < 0.01; ∗∗∗, *p* < 0.001). (**g**) Close-up view of the hydrophobic residues that yielded group (I) mutants (boxed residues) are shown as surface representations (left) and ribbon representations with sticks depicting side chains (right). The mutated residues are shown in bold. The distance between the Cβ atoms of F15 and Y32 is 9.4 Å, and that between F15 and F33 is 10 Å

To identify APETx1 residues that contribute to hERG inhibition, we selected 15 residues (T3, Y5, K8, F15, K18, T19, S22, N23, R24, T27, S29, Y32, F33, L34, and D42) for scanning mutational analysis (Fig. 3a-b), because the side chains of these residues are exposed on the molecular surface of APETx1 and thus are assumed to contribute to the hERG inhibition by making direct interactions with hERG. We designed alanine-substitution mutants, omitting T3. It has been reported that the natural T3P mutant, designated APETx3, does not inhibit hERG at a depolarization pulse of 40 mV [31], which prompted us to design a proline-substitution mutant. The ^1^H-^15^N HSQC spectra of all mutants are essentially identical to that of the wild-type (WT) APETx1; the exceptions to this are the mutated residue and a few neighboring residues (Fig. S5). This result indicates that none of the mutations affected the overall APETx1 structure.

hERG currents were measured in the presence or absence of the APETx1 mutants by whole-cell patch clamp recordings (Fig. 3c-f, Fig. S6). The toxin-induced *V*_1/2_ shift (Δ*V*_1/2_) value of 10 μM WT APETx1 was approximately 24 mV (Δ*V*_1/2_ = 23.9 ± 2.5 mV; Fig. 1d). The APETx1 mutants were categorized into three groups according to the Δ*V*_1/2_ values: (I) mutants that showed significantly decreased Δ*V*_1/2_ values relative to those of WT (F15A, Y32A, F33A, and L34A; Fig. 3c and f); (II) mutants exhibiting no significant change but a decreasing tendency in Δ*V*_1/2_ values relative to those of WT (K18A and T19A; Fig. 3d and f); and (III) mutants showing Δ*V*_1/2_ values nearly equal to those of WT (T3P, Y5A, F15A, S22A, N23A, T27A, S29A, and D42A; Fig. 3e-f). These results clearly indicate that F15, Y32, F33, and L34 play key roles in hERG inhibition. These four residues are localized on the molecular surface of APETx1, while K18 and T19, which yielded group (II) mutants, lie on the periphery of the group (I) site (Fig. 3g). In contrast to these two groups, the residues that yielded group (III) mutants were not located close to the four key residues but were instead dispersed on the molecular surface of APETx1 (Fig. 3b). These results suggest that the molecular surface formed by the residues F15, Y32, F33, and L34 are foundational to the interactions between APETx1 and hERG.

As noted above, APETx3 is reported to show no inhibitory effect on hERG [31]. Unexpectedly, our results showed that recombinant APETx3 shifted the *V*_1/2_ values toward positive voltage by approximately 19 mV, which is comparable to the effect of APETx1 (Fig. 3f). Note, a *V*_1/2_-shift effect has not been characterized for the native APETx3. Moreover, since the structure of native APETx3 is not reported, we are unable to confirm whether the recombinant APETx3 is structurally identical to native APETx3. Nonetheless, the chemical shift values of the recombinant APETx1 and APETx3 toxins are similar (Fig. S5), suggesting that the T3 position of APETx1 is not crucial for hERG inhibition.

### Mutations of hERG residues that affect inhibitory activity of APETx1

**Fig. 4:**
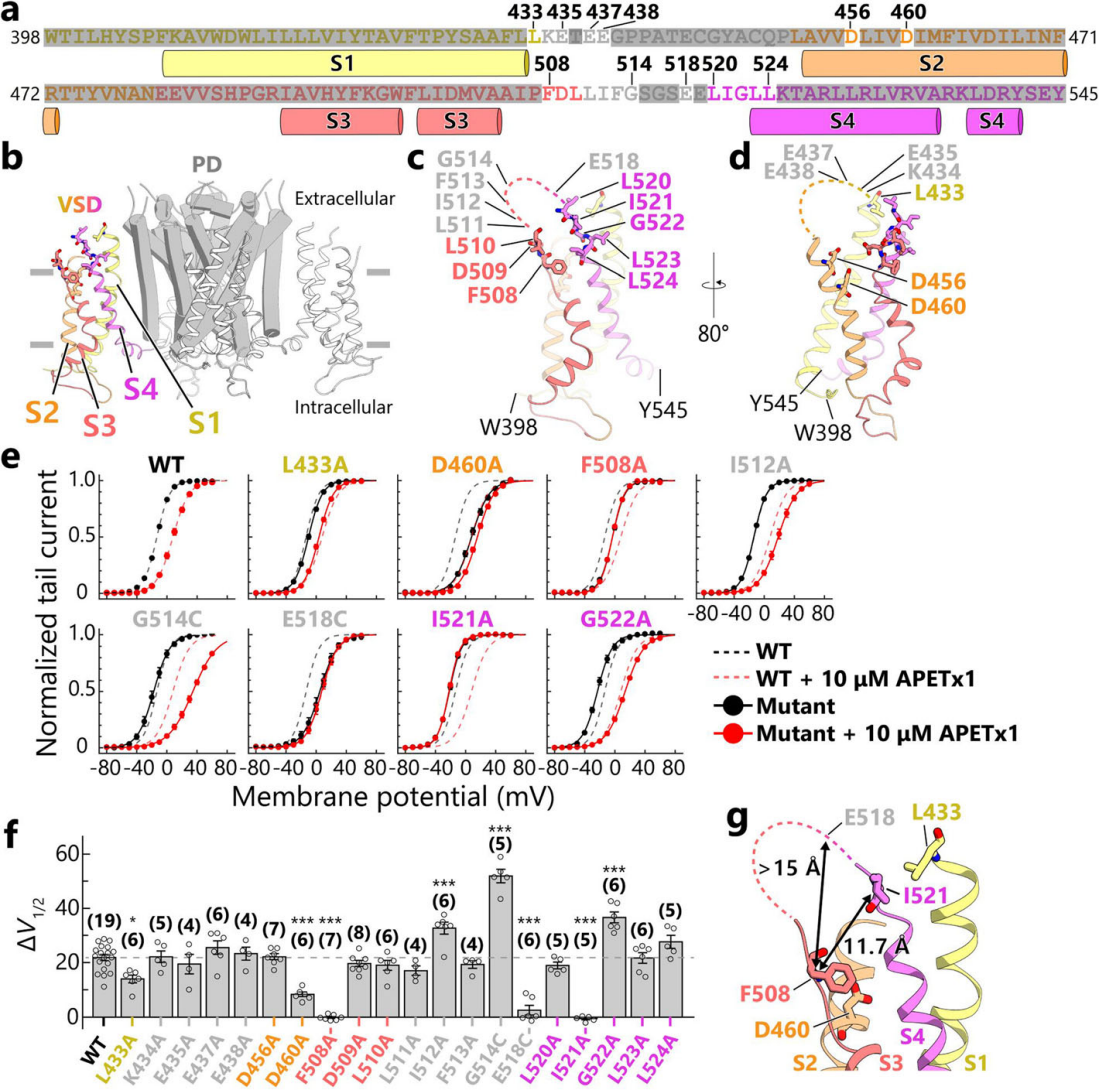
Inhibition of hERG mutants by APETx1. **a** The primary structure of the hERG VSD, colored as follows: S1, yellow; S2, orange; S3, pale red; and S4, magenta. The residues that are missing in a cryo-EM structure [17] are colored with gray. Non-mutated residues are drawn with semi-transparent gray marker to highlight the mutated residues. **b** The transmembrane domain of the cryo-EM structure of hERG with S4-up conformation (PDB code: 5VA2) [17]. The VSDs are represented as ribbons, one of which is colored as in (**a**); the others are white. The PD is represented as a gray cartoon. **c**, **d** A detailed illustration of the hERG VSD, corresponding to the sequence shown in (**a**). The mutated residues are represented as sticks. The residues in the extracellular region that are missing in the cryo-EM structure of hERG are depicted as dashed lines. **e** Normalized *G*-*V* curves (mean ± SEM) of hERG mutants showing Δ*V*_1/2_ values that are significantly different from those of WT, in the absence (black solid line) or presence (red solid line) of 10 μM APETx1. The fitting curves of WT in the presence (black dashed line) and absence (red dashed line) of 10 μM APETx1 are superimposed on those of the mutants. **f** The Δ*V*_1/2_ values of 10 μM APETx1 in hERG-mutants are represented as the mean values ± SEM; the number of experiments is shown in parentheses. Multiple-group comparison was performed by one-way ANOVA followed by Tukey’s test (∗, 0.01 ≤ *p* < 0.05; ∗∗, 0.001 ≤ *p* < 0.01; ∗∗∗, *p* < 0.001). **g** Close-up view of the residues that yielded mutations that decreased the hERG inhibition by APETx1. The distance between the Cβ atoms of F508 and I521 are 11.7 Å, and the distance between F508 and E518 is at least 15 Å

We introduced a mutation to hERG to investigate whether this would affect the inhibitory activity of APETx1 and thereby identify the APETx1-binding site on hERG. We proceeded based on the generally accepted proposal that the extracellular side of the VSD of a voltage-gated ion channel is targeted by gating-modifier toxins [32]. A previous study examining the APETx1-hERG interaction via cysteine-scanning mutational analysis of G514-E519, which are located in the S3-S4 region of the VSD, showed that G514C and E518C mutations respectively increased and decreased the Δ*V*_1/2_ value of 10 μM APETx1 [28]. These results suggest that these hERG residues in the S3-S4 region are involved in APETx1 binding [28]. As mentioned above, APETx1 shifted the *V*_1/2_ values toward positive voltage (Fig. 1c-d and Fig. S4c-d), which supports the possibility that APETx1 preferentially binds to hERG in its resting state, as described previously [27, 28]. However, it remains unknown where and how APETx1 interacts with the VSD to stabilize the resting state, S4-down conformation of hERG. Therefore, in the present study, we conducted a mutational analysis of the residues in the S3-S4 region of hERG, focusing on F508-L524, and omitting S515-S517 and E519, which have been previously investigated (Fig. 4a-c) [28]. We also examined the mutational effect of L433 and the charged residues (K434, E435, E437, E438, D456, and D460) on the S1-S2 region to confirm whether the S1-S2 region contributes to hERG inhibition of APETx1 (Fig. 4a-b and d).

We measured the currents of the hERG mutants by TEVC recordings using oocytes from *Xenopus laevis*. All mutants expressed in *X. laevis* oocytes showed hooked tail currents, which are characteristic features of hERG (Figs. S7, S8, S9 and S10) [3]. This demonstrates that the overall structures of hERG mutants are not substantially different from those of WT. It should be noted that, even in the absence of APETx1, some mutants (D456A, D460A, D509A, E518C, and L524A) exhibited *V*_1/2_ shifts toward positive voltage and others (L520A and L523A) toward negative voltage (Figs. S7, S8, S9 and S10). Therefore, the results pertaining to these mutants should be carefully evaluated.

We found that the Δ*V*_1/2_ values of 10 μM APETx1 in TEVC recordings were comparable to those in patch-clamp recordings in WT hERG (Δ*V*_1/2_ = 21.9 ± 1.1 mV, in TEVC recordings; Δ*V*_1/2_ = 23.9 ± 2.5 mV, in patch-clamp recordings; Figs. 1d and 4e). We confirmed that the Δ*V*_1/2_ values were increased by the G514C mutation and decreased by the E518C mutation (Fig. 4f), which is consistent with the results of a previous study [28]. We further found that the F508A and I521A mutations significantly decreased the Δ*V*_1/2_ values relative to WT, while the I512A and G522A mutations increased the values (Fig. 4c and e-f). Furthermore, mutations of L433 on S1 and D460 on S2 also significantly decreased the Δ*V*_1/2_ values relative to WT (Fig. 4d-f). These results suggest that the S3-S4 loop plays a key role in APETx1 binding, while L433 on S1 and D460 on S2 are also involved in the hERG inhibition of APETx1.

As mentioned above, the F508, E518, and I521 mutations in the hERG S3-S4 region appear to decrease the apparent binding affinity of APETx1 (Fig. 4f). Of these three key mutants, it is particularly important that we interpret the toxin-induced shifts in the activation voltage of the E518C mutant, as it demonstrated strongly positive shifts in activation voltage even in the absence of APETx1 (WT, *V*_1/2_ = − 13.0 ± 0.4 mV; E518C, *V*_1/2_ = 5.0 ± 2.7; Fig. S9). Meanwhile, L524A, another hERG mutation, also caused a large positive shift in activation voltage (L524A, *V*_1/2_ = 8.3 ± 1.5; Fig. S10). However, APETx1 shifted the activation voltage of the L524A mutant to the same extent as WT hERG. Alternatively, L520A and L523A caused negative shifts in activation voltage (L520A, *V*_1/2_ = − 34.4 ± 0.6 mV; L523A, *V*_1/2_ = − 40.8 ± 1.2 mV; Fig. S10). However, these two mutations did not alter the Δ*V*_1/2_ values of APETx1. Therefore, we considered that the large perturbation of gating states in hERG activation by mutations did not significantly affect the Δ*V*_1/2_ values of APETx1. These results suggest that the remarkable decrease in the APETx1 Δ*V*_1/2_ value by the hERG E518C mutation is not only attributed to a consequence of changing the channel gating properties, but also reduced toxin-binding.

### Construction of the docking models of APETx1-VSD complex that satisfy the electrophysiological results

To investigate whether or not any hypothetical structure would satisfy the results of the mutational analysis, we assumed that the key residues, inferred from the mutational analysis, are involved in the direct interaction of the toxin-channel complex. The Cβ atoms of the four key hydrophobic residues (F15, Y32, F33, and L34) of APETx1 are all located within 10 Å of each other (Fig. 3g), suggesting that the interacting counterpart residues are complementarily distributed on the hERG molecular surface. In the hERG S4-up conformation, previously revealed by cryo-EM analysis [17], the distance between the Cβ atoms of F508 and I521 is approximately 12 Å, while that between F508 and E518 appears to be greater than 15 Å (Fig. 4g); such distances are not complementary to the distribution of the key APETx1 residues. However, a downward S4-movement of hERG VSD may cause E518 and I521 on S4 to move closer to F508 on S3. This idea inspired us to investigate complementarity between APETx1 and hERG VSD in the S4-down conformation.

Visualization of hERG VSD in the S4-down conformation possesses two potential limitations. First, the coordinates of the hERG S3-S4 loop (L511–E519), which may be included in the APETx1-binding site, are missing in the cryo-EM structure and are unavailable [17]. Second, the structures of K_V_ channels with S4-down conformation have not been previously reported. To circumvent these challenges, we focused on the cryo-EM structure of the closely related K_V_ channel, rat *ether-à-go-go* potassium channel 1 (rEAG1), which includes the full coordinates of S3-S4 loop [21]. In the rEAG1 structure, the VSDs are uncoupled with the pore in the presence of Ca^2+^-calmodulin, and the structures of the pore in hERG and rEAG1 differ in open or closed gate [21]. However, the VSDs in both structures form similar S4-up conformations (Fig. S11a). Therefore, we used the structural model of rEAG1 as a template [21] for the construction of an S4-up model (“up” model, Fig. S11b). We further constructed two hypothetical S4-down models, the “one-helical-turn down” model and the “two-helical-turn down” model (Fig. S11b), in which the S4 helix is shifted toward the intracellular side by one or two helical turns, respectively. These S4-down models are based on the premise that in response to membrane repolarization, the S4 helix moves toward the intracellular side, with the associated basic residues maintaining salt bridges with the acidic residues in the S1-S3 helices [24–26, 33, 34]. Functional analysis suggests that the scheme of the gating-charge transfer in hERG activation is similar to that of most-studied Shaker-type K_V_ channels, in which the S4 helix moves to a roughly perpendicular position [35]. Thus, we used these simplified S4-down models to verify where the key hERG residues, F508, E518, and I521, become positioned following downward movement of the S4 helix.

In these models, the side chains of hERG E518 and I521 are exposed and constitute the largest surface areas in the S4-down models (Fig. S11e and g), which is in contrast to the S4-up model (Fig. S11c). The S4-down models show that the key hERG residues (F508, E518 and I521) are localized and form a crevice on the molecular surfaces, which appears complementary to the distribution of the APETx1 active residues (Fig. S11f and h, left, white dotted circle). By contrast, the S4-up model shows that the key hERG residues are not localized on the molecular surface, nor is a crevice formed, suggesting that all key APETx1 residues cannot simultaneously interact with the hERG S4-up conformation (Fig. S11d).

**Fig. 5:**
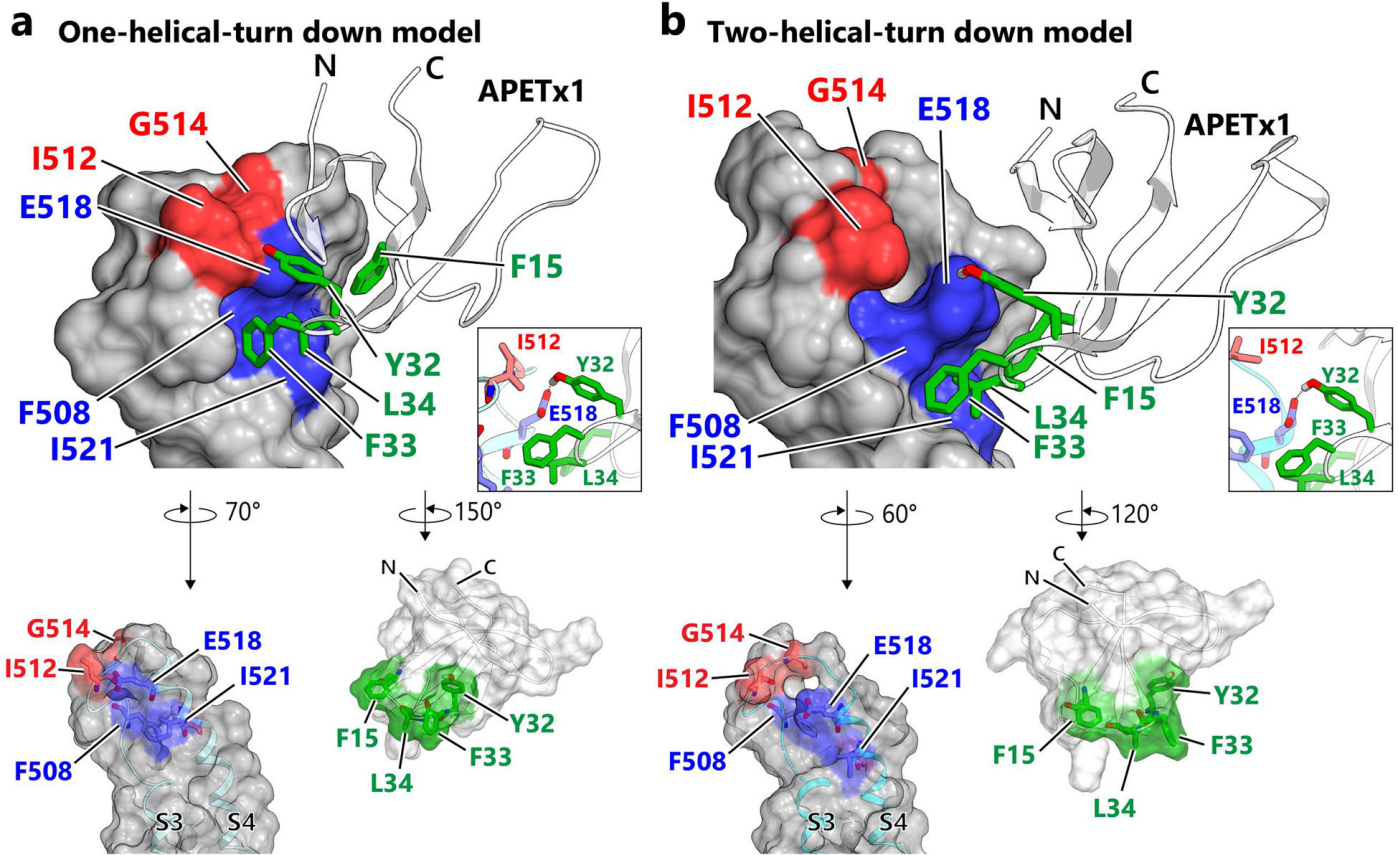
Structural models of the APETx1-VSD complex that satisfies the results of the mutational analysis. APETx1 docked to the one- and two-helical-turn-down model of hERG VSD in (**a**) and (**b**), respectively. The models exclude the S1 and S2 regions, which do not come into contact with APETx1, for clarity. The models of the hERG S3-S4 regions are represented as molecular surfaces colored according to the residues that yielded mutations that decreased (blue) and increased (red) inhibition by APETx1. APETx1 is represented as a ribbon with green sticks representing the key residues involved in hERG inhibition. Close-up view of the hydrogen bond between APETx1 Y32 and hERG E518 is shown in inset. “Open-book” representations of the interaction interfaces are also drawn as semi-transparent molecular surfaces with ribbon representation

Next, we docked APETx1 on the two S4-down models based on the assumptions that the key residues, identified by the mutational analyses of APETx1 and hERG, directly interact with each other (Fig. 5). Note, the APETx1-binding position on S3 and S4 of the hERG VSD face the lipid interface; thus, when bound to the hERG VSD, APETx1 is reasonably accommodated on S4, without contacting the PD or other VSDs (Fig. S12a-b). We also performed a docking simulation of APETx1 on the S4-up model. However, multiple clusters of docking models with no noticeable interaction of key residues were observed, making it difficult to select which model to compare to the S4-down models (data not shown).

Figure 5 shows the mapping of the residues that are key to hERG inhibition by APETx1 (see also Fig. S11c-h). On the S4-down models, a crevice formed by F508, E518, and I521 accommodates the F15, F33, and L34 side chains of APETx1 (Fig. S11f and h). Not only are the distributions of these residues on each molecule complementary in these models, but so too are the shapes of the molecular surfaces. These interactions were primarily formed by the hydrophobic residues of APETx1 and hERG. Outside the crevice formed in these models, the side chains between APETx1 Y32 and hERG E518 were positioned closely to allow for the potential formation of a hydrogen bond (Fig. 5a-b, inset).

APETx1 showed stronger inhibition of the hERG I512A mutant than WT (Fig. 4f). In the one-helical-turn down model, the sterically bulky I512 side chain lies in close proximity to E518 (Fig. 5a, inset), suggesting that I512 hinders access of APETx1 to E518 in the S4-down conformation. Hence, the increased inhibition of the I512A mutant by APETx1 could be due to the removal of steric hindrance, resulting in a higher binding affinity to the S4-down conformation. Moreover, the glycine residue mutants of hERG, G514C [28] and G522A, exhibit increased inhibition by APETx1 (Fig. 4f). These remarkable mutational effects might be due to the structural changes caused by the mutation of glycine residues adjacent to the critical residues (e.g., I512 and E518), which optimizes the contact surface for APETx1 binding.

## Discussion

### Structure and function of the recombinant APETx1

In the present study, we conducted structural and electrophysiological analyses of recombinant APETx1 and its mutants. We concluded that it is structurally identical to the natural product based on the chemical shift values of each under identical conditions (pH 3.0, 280 K; Fig. S3e). The structure of the C-terminal region determined in the present study at pH 6.0 and 298 K was slightly different from that of the natural product determined in a previous study under different conditions (pH 3.0, 280 K) [29]. We attribute this difference to variations in pH (Fig. S3f), and we used ^1^H-^15^N HSQC spectra to confirm that this structural change is reversible.

Recombinant APETx1 effectively inhibited the hERG currents elicited by relatively weak depolarization pulses (Fig. 1a), shifting the *V*_1/2_ values toward positive voltage in a dose-dependent manner (Fig. 1c-d, Fig. S4c-d). This positive *V*_1/2_-shift effect suggests that APETx1 recognizes and stabilizes the resting state of hERG, in which the VSD adopts the S4-down conformation, as described previously [27, 28]. It should be noted that APETx1 caused a reduction in the maximal conductance at higher voltages and faster attenuation of the tail currents (Fig. 1b and Fig. S7), suggesting that APETx1 remains hERG-bound even when strong depolarizing pulses are applied. These implicative phenomena were also reported in several other gating-modifier toxins that stabilize the S4-down conformation of voltage-gated ion channels [32, 36–43].

A previous study reported that an increase in Δ*V*_1/2_ values occurs at a micromolar concentration of APETx1 (10^− 6^ M), while the *K*_d_ values derived from *I*_toxin_ / *I*_control_ at weak depolarizing pulses are 10^− 7^–10^− 8^ M [28]. In our TEVC recordings, however, both a decrease in *I*_toxin_ / *I*_control_ (Fig. S4b) and an increase in the Δ*V*_1/2_ values (Fig. S4d) were observed at micromolar concentrations. The reasons for the differences between the present and previous studies remain unclear as we were unable to directly compare the recombinant APETx1 and the natural product in the same experimental systems.

### Hydrophobic surface of APETx1 contributes to hERG inhibition

We established a method for the preparation of recombinant APETx1, which enabled us to conduct mutational analysis. The electrophysiological analysis using the APETx1 mutants clearly showed that the four hydrophobic residues (F15, Y32, F33, and L34) play pivotal roles in hERG inhibition. Two hydrophilic residues, K18 and T19, also appear to contribute to the inhibition. By mapping these residues onto the APETx1 structure, we revealed that they are localized on the APETx1 molecular surface (Fig. 3g), where the four hydrophobic residues are clustered at the edge of the large hydrophobic surface; this is shown in green in Fig. 3g. It is well known that many gating-modifier toxins possess a large hydrophobic surface, called a hydrophobic patch, which is reported to play a role in partitioning the cell membrane prior to the binding of the target channel [44]. A spider gating-modifier toxin, SGTx1, uses the residues on the hydrophobic patch to interact with both the lipid membrane and the target channel [45, 46]. It has not been reported whether the hydrophobic patch on APETx1 contributes to such membrane-partitioning and allosteric inhibition through binding of membrane lipids. However, the drastic decreases observed in the Δ*V*_1/2_ values of the four hydrophobic residues mutations, along with their localization on the APETx1 surface, suggest that these residues engage in direct interactions with hERG in the membrane, which could stabilize the hERG resting state.

### Putative APETx1-binding residues of hERG

In the present study, we identified four previously undescribed hERG residues in the S3-S4 region (F508, I512, I521, and G522); mutations of these, along with the previously reported residues G514 and E518 [28], affect hERG inhibition of APETx1 (Fig. 4f). These results show that the structure of the S3-S4 region plays a key role in APETx1-binding.

A key hERG mutation, E518C, caused the large positive shifts in the hERG activation voltage (Fig. S9). Other mutations, L520A, L523A, and L524A, which perturb the gating state in hERG activation, have only minimal effects on the Δ*V*_1/2_ values of APETx1 (Fig. 4f and Fig. S10). Based on these results, we determined that E518 is likely the residue that is targeted by APETx1, as well as F508 and I521 in the present study.

Three hERG mutants, I512A, G514C, and G522A, were more susceptible to the inhibition by APETx1 than WT (Fig. 4f). These mutated residues are on the S3-S4 region and located near the key residues, F508, E518, and I521, which are involved in the inhibition by APETx1. The I512A mutation could reduce the steric bulkiness of the side chain of isoleucine in the position around the key residues. The mutations, G514C and G522A, would affect the local structure of the key residues. Therefore, these mutations increased the inhibition by APETx1, which supports the idea that APETx1 recognizes the S3-S4 region of hERG.

### Limitation of the APETx1-VSD complex models

One of the best ways to obtain pairwise information on the interacting residues between hERG and APETx1 is through thermodynamic mutant cycle analysis [47]. However, this analysis could not be conducted in the context of the hERG-APETx1 interaction because even a single mutation in either APETx1 or hERG decreases the Δ*V*_1/2_ values to nearly zero in the presence of 10 μM APETx1 or its mutant (Figs. 3f and 4f); thus, the additive effect of the double mutation cannot be evaluated.

Our docking models of the APETx1-VSD complex in the S4-down conformation were constructed to investigate whether any hypothetical structure would satisfy the results of the mutational analysis (Fig. 5a-b). These models are based on the assumption that the key residues identified in the mutational analysis interact with each other directly. Moreover, although the S4-movement in hERG activation is characterized by several functional analyses, the corresponding structural properties remain unclear [17, 35, 48]. We therefore constructed simplified S4-down models by shifting one or two helical turns downward. Although, we concede that it is not possible to determine which model is favorable using only calculated parameters, such as buried surface areas, the models constructed here seem to satisfy our mutational results. Further experimental validation and molecular dynamics simulations are required to verify these models.

In the present study, recombinant APETx3 showed a Δ*V*_1/2_ value comparable to that of APETx1 (Fig. 3f). Although direct comparison between the recombinant and native APETx3 is required to reveal whether this result can be applied to the native APETx3, we have successfully demonstrated that T3 of APETx1 has little involvement in hERG inhibition, which is consistent with our model, in which T3 of APETx1 does not directly contact the hERG VSD.

The mutational analysis of hERG clearly shows that the S3-S4 region is crucial for inhibition by APETx1, but we cannot rule out the possibility that an additional hERG region is also involved in binding with APETx1. In particular, the mutations of L433 on S1 and D460 on S2 decreased inhibition by APETx1 (Fig. 4f-g). Although these residues do not make direct contact with APETx1 in our models (Fig. S12a-b), their mutations might affect the structure of the APETx1-binding site in the S3-S4 region.

## Conclusions

The present study identified the key residues of APETx1 and hERG that are involved in hERG inhibition by APETx1. Based on the assumption that the distribution of these residues on each molecule is complementary, we built structural models of APETx1-VSD complex that satisfy the electrophysiological results. The identified key residues will help advance understanding of the inhibitory mechanism of APETx1 and other gating-modifier toxins, which could provide a structural basis for the creation of novel types of drugs targeting the VSDs of K_V_ channels in the future.

## Methods

### Recombinant expression, purification, and refolding of WT and mutated APETx1

The DNA sequence encoding APETx1, along with an upstream TEV-protease-recognition sequence, were cloned into the pET-30Xa/LIC vector (Novagen). All APETx1 mutants were generated by PCR-mediated site-directed mutagenesis and confirmed by DNA sequence analysis.

The plasmids were transformed into *Escherichia coli* strain C41 (λDE3) for recombinant protein production. The cells were grown in Luria-Bertani medium supplemented with 40 mg/mL kanamycin and maintained at 37 °C with shaking at 150 rpm to an optical density of 0.6–1.0 at 600 nm. Expression of the His_6_-tag-fusion proteins was induced with 1 mM isopropyl β-D-1-thiogalactopyranoside (IPTG); the cells were grown for 6–12 h at 37 °C and then harvested by centrifugation for 10 min at 5000×g. Uniformly ^15^N-labelled or uniformly ^13^C- and ^15^N-labelled APETx1 samples were prepared for NMR experiments by growing *E. coli* cells in M9 minimal medium supplemented with ^15^NH_4_Cl or ^15^NH_4_Cl and ^13^C_6_ glucose.

The cells were harvested by centrifugation and disrupted by sonication in lysis buffer (20 mM Tris-HCl, pH 8.0, and 200 mM NaCl) supplemented with 0.5 mM 4-(2-aminoethyl)benzenesulfonyl fluoride hydrochloride, 0.15 μM aprotinin, 1 μM E-64, and 1 μM leupeptin. The cell pellets were prepared by centrifugation for 30 min at 10000×g. The pellets were resuspended in lysis buffer containing 0.1% Nonidet P-40, followed by centrifugation; this procedure was repeated three times for the removal of nucleic acid. The pellets were solubilized using the lysis buffer containing 8.0 M urea for 1–2 h at room temperature (25 °C–27 °C). After centrifugation for 30 min at 10000×g, the His_6_-tag-fusion proteins were purified from the supernatant by a HIS-Select® Nickel Affinity Gel (Sigma) column. The eluted His_6_-tag-fusion proteins were reduced by 2 mM dithiothreitol (DTT), followed by dilution to 3 μM or less so as to avert aggregation during the subsequent refolding step. The reduced proteins were refolded by dialysis against a redox buffer (3 mM reduced glutathione, 0.3 mM oxidized glutathione, 10% glycerol, 20 mM Tris-HCl, pH 9.0, and 200 mM NaCl) until the concentration of urea was less than 200 mM. This internal solution was next dialyzed against a refolding buffer (20 mM Tris-HCl, pH 9.0, and 200 mM NaCl) until the urea concentration was less than 10 mM. The internal solution was concentrated using Sep-Pak® C18 cartridges (Waters), and then lyophilized. The sample was dissolved in lysis buffer and then centrifuged to remove the aggregated proteins, which form intermolecular disulfide bonds. After digestion with His-tag-fused TEV protease at 25 °C, cleaved His-tag and His-tag-fused TEV protease were removed using a HIS-Select® Nickel Affinity Gel (Sigma-Aldrich) column. APETx1 was further purified by reverse-phase high-performance liquid chromatography (RP-HPLC) using an ODS-AM column (YMC). A linear gradient from 20 to 50% acetonitrile at a flow rate of 5.0 mL/min for 30 min was performed using water and acetonitrile containing 0.1% trifluoroacetic acid (TFA). The eluted APETx1 was detected by matrix-assisted laser-desorption ionization–time-of-flight mass spectroscopy (MALDI-TOF MS) on a MALDI-8020 mass spectrometer (Shimadzu). α-cyano-4-hydroxycinnamic acid was used for the matrix. An external calibration was performed using a ProteoMass Peptide and Protein MALDI-MS Calibration Kit (Sigma-Aldrich).

### NMR resonance assignments of APETx1 and validation of mutants

Data were collected using Bruker Avance 500 or 600 spectrometers equipped with triple-resonance probes. For NMR resonance assignments of APETx1, we measured ^1^H-^15^N HSQC spectra, ^1^H-^13^C HSQC spectra, and non-uniformly sampled, three-dimensional NMR spectra using 258 μM uniformly ^13^C- and ^15^N-labelled APETx1 in a buffer containing 20 mM KH_2_PO_4_ (pH 6.0), 100 mM NaCl, and 10% D_2_O (hereafter referred to as “phosphate buffer”) at 298 K. The mixing times of the ^15^N-edited and the ^13^C-edited NOESY (nuclear Overhauser effect spectroscopy) experiments, which were used for sequential assignments, were set to 100 ms and 120 ms, respectively. ^1^H-^15^N HSQC spectra were also measured at different pH levels (pH 3.0, pH 4.5, and pH 6.0; the phosphate buffer was pH-adjusted with HCl) and temperatures (280 K, 290 K, and 298 K) using 140 μM uniformly ^15^N-labelled APETx1.

The ^1^H-^15^N HSQC spectra of uniformly ^15^N-labelled WT APETx1 and selected mutants (T3P, K8A, F15A, K18A, R24A, Y32A, and F33A) were measured in the phosphate buffer at 298 K; those of WT and the remaining mutants (Y5A, T19A, S22A, N23A, T27A, S29A, L34A, and D42A) were measured in 10% D_2_O (pH 6.0) at 298 K. Sample concentrations of WT and mutants are as follows: WT, 149 μM (in the phosphate buffer) or 102 μM (in 10% D_2_O, pH 6.0); T3P, 50 μM; Y5A, 229 μM; K8A, 30 μM; F15A, 184 μM; K18A, 31 μM; T19A, 290 μM; S22A, 102 μM; N23A, 76 μM; R24A, 19 μM; T27A, 129 μM; S29A, 86 μM; Y32A, 408 μM; F33A, 634 μM; L34A, 258 μM; and D42A, 174 μM.

All spectra were processed using Bruker TopSpin 3.6 software or NMRPipe [49], and the data were analyzed with Sparky (T. D. Goddard and D. G. Kneller, Sparky 3, University of California, San Francisco, CA). The APETx1 backbone and side-chain NMR signals were sequentially assigned using non-uniformly sampled data for the following experiments: HNCACB, CBCA(CO)NH, HNCO, HCCH-COSY (correlation spectroscopy), HCCH-TOCSY (total correlation spectroscopy), ^15^N-edited TOCSY, ^15^N-edited NOESY, and ^13^C-edited NOESY experiments. The amide signals of T2 and L34 were not sequentially assigned due to the absence of these signals on three-dimensional triple-resonance NMR spectra at pH 6.0; however, they were observed on ^1^H-^15^N HSQC spectra under low pH conditions (pH 3.0 and pH 4.5 at 298 K). ^1^H chemical shift values of WT APETx1 were obtained using sodium 4,4-dimethyl-4-silapentane-1-sulfonate (DSS) as a standard; ^13^C and ^15^N chemical shift values were also corrected indirectly. The ^1^H, ^13^C, and ^15^N chemical shift assignments at pH 6.0 and 298 K have been deposited in Biological Magnetic Resonance Bank (BMRB ID: 36345).

### NMR structure calculation of APETx1

Data were collected using Bruker Avance III HD 700 spectrometers equipped with triple-resonance cryogenic probes at 298 K. All experiments were performed using 691 μM uniformly ^13^C/^15^N-labelled APETx1 in a buffer solution containing 20 mM KH_2_PO_4_ (pH 6.0), 100 mM NaCl, and 10% D_2_O. The mixing times for the ^15^N-edited and the ^13^C-edited NOESY experiments for structural determination were set to 200 ms. These spectra were processed using NMRPipe [49].

Based on the chemical shift difference between Cβ and Cγ [50], we confirmed that the peptide bond of the P40 residue is *cis*-conformer, which is consistent with the structure of the natural product [29]. This *cis*-peptide restraint was used for structure calculation. The dihedral angle restraints were predicted using TALOS+ and were based on the chemical shifts of ^13^Cα, ^13^Cβ, ^13^CO, ^15^Nα, and ^1^HN [51]. NOE peaks were automatically picked using MagRO-NMRView [52–54]. The NOE peak intensity was converted to distance constraints, and structure calculation was performed using the torsion angle dynamics program CYANA 3.98 [55]. First, we calculated the preliminary structures without disulfide bond restraints to confirm that three pairs of cysteine residues can be correctly formed into disulfide bonds. Next, we calculated the structures using disulfide bond restraints and obtained 100 structures in the final iteration. The 20 structures with the lowest target function were refined by restrained molecular dynamics of 30 ps with Amber 12 [56]. The automated identification and superposition of the ordered regions of the determined structures were performed by using FitRobot ver. 1.00.07 [57]. Atomic coordinates of APETx1 and all restraint files used for the structure calculations have been deposited in the Protein Data Bank (PDB code: 7BWI). NMR structure ensembles were visualized and the root mean square deviation (RMSD) values were calculated using MOLMOL [30].

### Cell preparation for patch-clamp recordings

Stable HEK 293 cell lines expressing hERG (SB-HEK-hERG; SB Drug Discovery Limited) were used. The cells were cultured in Dulbecco’s Modified Eagle’s Medium (DMEM; Thermo, Gibco) supplemented with 10% fetal bovine serum (FBS; Thermo, Gibco) and 1% penicillin/streptomycin (Thermo, Gibco) in a humidified 5% CO_2_ atmosphere at 37 °C. For patch-clamp experiments, cultured cells on a polystyrene culture dish (Sumitomo Bakelite, Tokyo, Japan) were detached by TrypLE Express (Thermo, Gibco) at 37 °C.

### Automated patch-clamp recordings for mutational analysis of APETx1

Whole-cell automated patch-clamp recordings were obtained using a SyncroPatch 384PE (Nanion Technologies GmbH, Germany) with single-hole medium resistance (4–5.5 MΩ) borosilicate glass planar chips. Pulse generation and data collection were performed with PatchControl 384 V1.6.6 and DataControl384 V1.8.0 software. Currents were sampled at 1 kHz. Leak subtraction was performed based on a small voltage step at the beginning of the voltage protocol. Seal resistance was calculated using built-in protocols, and cells with a seal resistance of 0.3–3 GΩ were analyzed.

For automated patch-clamp recordings, the intracellular solution contained 110 mM KF, 10 mM NaCl, 10 mM KCl, 10 mM EGTA, and 10 mM HEPES-KOH (pH 7.2); the extracellular solution contained 140 mM NaCl, 4 mM KCl, 2 mM CaCl_2_, 1 mM MgCl_2_, 5 mM D-glucose, and 10 mM HEPES-NaOH (pH 7.4). The command voltage step took into account the fact that the use of these solutions results in ~ 9 mV liquid junction potential. All experiments were performed at room temperature (20 °C–25 °C). The holding membrane potential was set at − 80 mV. For the conductance-voltage (*G*-*V*) relationship experiments, hERG currents were elicited by depolarizing voltage steps from − 80 mV to + 60 mV in 10 mV increments for 2 s, followed by a step pulse to − 40 mV for 2 s.

In order to determine the dose-response relationship, APETx1 was added to the extracellular solution at concentrations of 0.1–10 μM. APETx1 mutants were added to the extracellular solution at 10 μM for screening. The voltage-pulse protocol for determining the *G*-*V* relationship was performed after the effects of APETx1 WT or mutants reached a steady state.

### Ethical approval

All animal experiments were approved by the Animal Care Committee of the National Institutes of Natural Sciences (an umbrella institution of National Institute for Physiological Sciences, Tokyo, Japan), and were performed in accordance with its guidelines.

### Source of animal

*Xenopus laevis* were purchased from Hamamatsu Animal Supply Co. (Hamamatsu, Japan) and used for oocyte collection.

### Preparation for TEVC recordings

hERG was subcloned into an pSP64 plasmid, and hERG mutants were generated by PCR-mediated site-directed mutagenesis using an In-Fusion HD Cloning Kit (Takara, Otsu, Japan) as described previously [58]. The DNA sequences of all mutants were confirmed by sequencing. The complementary RNAs (cRNA) were transcribed from each linearized plasmid DNA using the mMessage mMachine SP6 Transcription Kit (Ambion, Austin, TX, USA). *Xenopus laevis* were purchased from Hamamatsu Animal Supply Co. (Hamamatsu, Japan) and used for oocyte collection. Preparation and injection of *X. laevis* oocytes were performed as described previously [58, 59]. The total number of frogs for collecting oocytes in the present study was ~ 20. Frogs were anesthetized by 0.15% tricaine during surgery. After depletion of oocytes, anaesthetized frogs were killed by double pithing. Currents were measured 1–3 d after the cRNA injection, depending on the required current amplitude.

### TEVC recordings for mutational analysis of hERG

TEVC recordings were performed essentially as described previously [58, 59]. Macroscopic currents were recorded from injected oocytes under a two-electrode voltage clamp (TEVC) using an amplifier (OC-725C; Warner Instruments, Hamden, CT, USA), an AD-DA converter (Digidata version 1440A; Molecular Devices, Sunnyvale, CA, USA), and software for control and recording of the voltage clamp (pCLAMP version 10.7; Molecular Devices). Glass microelectrodes were drawn from borosilicate glass capillaries (Harvard Apparatus, Cambridge, MA, USA) and filled with 3 M potassium acetate and 10 mM KCl, pH 7.4 adjusted with HCl). The electrode resistance was 0.2–0.8 MΩ. Oocytes expressing cysteine-substitution mutants (G514C and E518C) were incubated in 10 mM DTT-containing frog Ringer’s solution (88 mM NaCl, 1 mM KCl, 2.4 mM NaHCO_3_, and 0.3 mM Ca(NO_3_)_2_, 0.41 mM CaCl_2_, 0.82 mM MgSO_4_, and 15 mM HEPES-NaOH, pH 7.4 with 0.1% penicillin-streptomycin) at 20 °C–25 °C for 15 min, and were thoroughly washed with DTT-free Ringer’s solution. All measurements were performed at 20 °C–25 °C and conducted within 30 min so as to avoid the formation of nonspecific disulfide bonds [28, 60]. The bath solution was ND96 (96 mM NaCl, 2 mM KCl, 1.8 mM CaCl_2_,1 mM MgCl_2_, and 5 mM HEPES-NaOH, pH 7.4). The holding membrane potential was set at − 90 mV. For hERG *G*-*V* relationship experiments (WT and mutants except for L520A and L523A), currents were elicited by depolarizing voltage step pulses from − 80 mV to + 60 mV in 10-mV increments for 1 s, followed by a step pulse to − 60 mV for 1 s. Due to the negatively shifted activation voltage of L520A and L523A, the magnitude of the depolarizing step pulses was individually adjusted for each of these mutants.

To determine the *K*_d_ value of APETx1 binding to hERG, the initial APETx1 concentration in the bath solution of 0.1 μM, and the concentration was increased cumulatively up to 10 μM. For mutational screening of hERG, APETx1 was added to the bath solution at a concentration of 10 μM, which is maximal concentration that oocytes can tolerate without leakage. The voltage-pulse protocol for *G*-*V* relationships was performed after the effect of APETx1 reached a steady state.

### Analysis of *G*-*V* relationships

All curve-fittings were performed using MATLAB R2019b (MathWorks Inc., Natick, MA). The *G*-*V* curves were fitted with the Boltzmann function:

$$ G\kern0.5em /\kern0.5em {G}_{\mathrm{max}}=1/1\left(1+{\mathrm{e}}^{\left({V}_{1/2}-\kern0.5em V\right)\kern0.5em /k}\right)+C $$

where the *G* / *G*_max_ is measured conductance relative to the maximal conductance, as determined from the peak of the outward tail current at − 40 mV in TEVC recordings or at − 60 mV in patch-clamp recordings; the *V*_1/2_ value is the membrane potential when the *G*-*V* relationship reaches half-maximal activation; the *k* value is the slope factor; and *C* is a constant component.

### Statistical analysis

All averaged data are presented as the mean ± SEM. The n value is the number of recordings. Multiple-group comparison was performed by one-way analysis of variance (ANOVA) followed by Tukey’s test using IBM SPSS Statistics, Version 25 (IBM Corp. Armonk, NY.). *p* < 0.05 was considered statistically significant (∗,0.01 ≤ *p* < 0.05; ∗∗, 0.001 ≤ *p* < 0.01; ∗∗∗, *p* < 0.001).

### Construction of the structural model of the APETx1-VSD complex

Homology models of the hERG VSD (residues 398–545) showing the S4-up or S4-down conformation were built using MODELLER 9.23 [61]. The cryo-EM structure of rEAG1 (PDB code: 5K7L) [21] was utilized as a template. The intact sequence alignment was used for the up model, whereas the three- and six-residue downward shift sequence alignments of S4 were used for the one- and two-helical-turn down models, respectively.

The structural model of the APETx1-hERG complex was generated with the HADDOCK2.4 web server [62]. The unambiguous distance restraints were tabulated so that the key APETx1 residues (F15, Y32, F33, and L34) and the key hERG residues (F508, E518, and I521) would be located within 3.0 Å or less. The hydrogen bond restraints were also specified between the APETx1 Y32 and hERG E518 side chains. For rigid-body energy minimization, 1000 structures with the 200 lowest energy solutions were generated and used for subsequent semi-flexible simulated annealing and water refinement. Molecular graphics figures were depicted using CueMol (http://www.cuemol.org/).

## Supplementary Information

**Additional file 1: Figure S1.** Reverse-phase HPLC and MALDI-TOF MS analysis of recombinant APETx1. (a) Chromatogram (left y-axis, black solid line) and gradient protocol (right y-axis, blue dashed line). (b) MALDI-TOF mass spectrum.

**Additional file 2: Figure S2.** NMR resonance assignments of APETx1 on the ^1^H-^15^N HSQC and ^1^H-^13^C HSQC spectra observed at pH 6.0 and 298 K. ^1^H-^15^N HSQC spectrum (a), ^1^H-^13^C HSQC spectra for the aromatic region (b), and for the aliphatic region (c).

**Additional file 3: Figure S3.** Variable-temperature and pH-titration NMR measurements using ^1^H-^15^N HSQC. (a) Overlay of the ^1^H-^15^N HSQC spectra at pH 6.0 under the different temperature conditions. (b-d) Overlay of the ^1^H-^15^N HSQC spectra at 298 K, 290 K, and 280 K in (b), (c), and (d), respectively, under different pH conditions. (e) ^1^H chemical shift differences of the backbone amide protons between recombinant APETx1 from the present study (BMRB ID: 36345) and the natural product from a previous study (BMRB ID: 6370) [29] under the same conditions (pH 3.0 and 280 K). The ^1^H chemical shift values found in the previous study were subtracted from those found in the present study. The proline residues, which lack amide protons, are labeled in gray. Asterisks (*) show that the amide signal was not observed at pH 3.0 and 280 K. (f) pH-dependent chemical shift change (Δδ) values of the ^1^H-^15^N HSQC spectral signals at 298 K (b). The Δδ values were calculated using the following equation [63]: Δδ = [(Δδ_1H_)^2^ + (Δδ_15N_ / 6.5)^2^]^1/2^ where Δδ_1H_ and Δδ_15N_ are the chemical shift changes in the ^1^H and ^15^N dimensions, respectively. The proline residues, which lack amide protons, are labeled in gray. An asterisk (*) represents G1, which was not observed in the ^1^H-^15^N HSQC spectra.

**Additional file 4: Figure S4.** Dose-dependent effects of APETx1 on hERG in TEVC recordings. (a) *G-V* curves (mean ± SEM) of hERG in the presence or absence of different concentrations of APETx1. (b) Dose-response curve using the fraction of uninhibited currents at the depolarizing pulse, − 30 mV . We fitted the data with the following three models [28]: (A) four equivalent and independent binding sites per channel with fractional toxin-sensitive current, *I*_Toxin_ / *I*_control_ = *A*_max_ [*K*_d_ / (*K*_d_ + [APETx1])]^4^ + (1 − *A*_max_), *K*_d_ = 1.2 μM, *A*_max_ = 0.87; (B) Four equivalent and independent binding sites per channel with fully toxin-sensitive current, *I*_Toxin_ / *I*_control_ = [*K*_d_ / (*K*_d_ + [APETx1])]^4^, *K*_d_ = 1.7 μM; (C) One binding site per channel with fractional toxin-sensitive current, *I*_Toxin_ / *I*_control_ = *A*_max_ [*K*_d_ / (*K*_d_ + [APETx1])] + (1 − *A*_max_), *K*_d_ = 0.23 μM, *A*_max_ = 0.93. (c) Normalized *G-V* curves (mean ± SEM). (d) The Δ*V*_1/2_ values of different concentrations of APETx1. Data points and error bars represent the mean values ± SEM.

**Additional file 5: Figure S5.** ^1^H-^15^N HSQC spectra of APETx1 mutants. All ^1^H-^15^N HSQC spectra were measured at pH 6.0 and 298 K in the following solution: T3P, K8A, F15A, K18A, R24A, Y32A, or F33A mutants, 20 mM potassium phosphate (pH 6.0), 100 mM KCl, and 10% D_2_O; Y5A, T19A, S22A, N23A, T27A, S29A, L34A, or D42A mutants, 10% D_2_O (pH 3.0); and WT recorded in both conditions. The spectrum of each mutant is superimposed onto that of WT under identical solution conditions. NMR resonance assignments were labeled according to the signals of WT.

**Additional file 6: Figure S6.** *G*-*V* curves of hERG in the presence or absence of 10 μM APETx1 and mutants. Normalized *G*-*V* curves (mean ± SEM) of hERG in the presence of 15 APETx1 mutants are sorted by amino acid residue order, divided by three into the five panels.

**Additional file 7: Figure S7.** The current traces and *G*-*V* curves of hERG and its mutants in the presence or absence of 10 μM APETx1. Current traces of the hERG mutants before and after the administration of 10 μM APETx1 (left). Voltage protocol is illustrated at the bottom of each current trace. Current traces and voltage protocols at arbitrary potentials are depicted with red to clearly indicate the current reduction by the inhibitory effect of APETx1. G-V curves (mean ± SEM) of hERG mutants in the absence (black filled circle and solid line) or presence (red filled circle and solid line) of 10 μM APETx1 (right). The fitting curves of the WT in the absence (black dashed line) and presence (red dashed line) of 10 μM APETx1 are superimposed onto those of the mutants.

**Additional file 8: Figure S8.** The current traces and *G*-*V* curves of hERG mutants in the presence or absence of 10 μM APETx1. Current traces of the hERG mutants before and after the administration of 10 μM APETx1 (left). Voltage protocol is illustrated at the bottom of each current trace. Current traces and voltage protocols at arbitrary potentials are depicted with red to clearly indicate the current reduction by the inhibitory effect of APETx1. G-V curves (mean ± SEM) of hERG mutants in the absence (black filled circle and solid line) or presence (red filled circle and solid line) of 10 μM APETx1 (right). The fitting curves of the WT in the absence (black dashed line) and presence (red dashed line) of 10 μM APETx1 are superimposed onto those of the mutants.

**Additional file 9: Figure S9.** The current traces and *G*-*V* curves of hERG mutants in the presence or absence of 10 μM APETx1. Current traces of the hERG mutants before and after the administration of 10 μM APETx1 (left). Voltage protocol is illustrated at the bottom of each current trace. Current traces and voltage protocols at arbitrary potentials are depicted with red to clearly indicate the current reduction by the inhibitory effect of APETx1. G-V curves (mean ± SEM) of hERG mutants in the absence (black filled circle and solid line) or presence (red filled circle and solid line) of 10 μM APETx1 (right). The fitting curves of the WT in the absence (black dashed line) and presence (red dashed line) of 10 μM APETx1 are superimposed onto those of the mutants.

**Additional file 10: Figure S10.** The current traces and *G*-*V* curves of hERG mutants in the presence or absence of 10 μM APETx1. Current traces of the hERG mutants before and after the administration of 10 μM APETx1 (left). Voltage protocol is illustrated at the bottom of each current trace. Current traces and voltage protocols at arbitrary potentials are depicted with red to clearly indicate the current reduction by the inhibitory effect of APETx1. G-V curves (mean ± SEM) of hERG mutants in the absence (black filled circle and solid line) or presence (red filled circle and solid line) of 10 μM APETx1 (right). The fitting curves of the WT in the absence (black dashed line) and presence (red dashed line) of 10 μM APETx1 are superimposed onto those of the mutants.

**Additional file 11: Figure S11.** Model building of the APETx1-VSD complex. (a) Sequence alignment and stereo view of structure comparison between the VSD of hERG (The Universal Protein Resource Knowledgebase (UniProtKB) Entry: Q12809, PDB code: 5VA2) and that of rEAG1 (UniProtKB Entry: Q63472, PDB code: 5K7L). hERG is colored as in Fig. 4a, and rEAG1 appears dark gray. (b) Validation of the homology models of the hERG S3-S4 region. Ribbon representation of the cryo-EM structure of hERG (PDB code: 5VA2) [17], up model, one- and two-helical-turn down models (from left to right). Basic residues at positions K1-R5 on S4, and the gating charge transfer center residue (F463) and the acidic residues (D456, D460, and D466) on S2 are represented as sticks. To clearly show the position of S4, the Cα positions of the S2 residues are depicted with horizontal gray dashed (D456, D460, and D466) or solid lines (F463). (c) Ribbon and semi-transparent surface representations of the up model of the S3-S4 region, colored as in Fig. 5. (d) Close-up view of (c). The one- (e) and two-helical-turn down models (g) of the S3-S4 region are represented as ribbons and semi-transparent surfaces colored as in Fig. 5. On the left of (f) and (h) are close-up views of (e) and (g), respectively. White dotted circles indicate the crevice between F508, E518, and I521. On the right, docked APETx1 is also displayed as semi-transparent ribbons with the green sticks indicating key residues involved in hERG inhibition.

**Additional file 12: Figure S12.** The binding location of APETx1 in a tetrameric transmembrane architecture of hERG. APETx1-VSD complex models, in which S4 adopts one- and two-helical-turn down conformations in (a) and (b), respectively, are superimposed onto the transmembrane domain of the cryo-EM of hERG (PDB code: 5VA2) [17], viewed from within the membrane plane (left) and from the extracellular side (right). One of the four VSDs of the cryo-EM structure is substituted by the APETx1-VSD complex model. The PD is represented as a cartoon, the VSDs as ribbons, and APETx1 as tubes. In the APETx1-docked VSD, S1 is yellow; S2, orange; S3 and S4 in (a), sky blue; and S3 and S4 in (b), cyan.

## Data Availability

The ^1^H, ^13^C, and ^15^N chemical shift assignments have been deposited in Biological Magnetic Resonance Bank (BMRB ID: 36345). The atomic coordinates of APETx1 and all restraint files used for the structure calculations have been deposited in the Protein Data Bank (PDB code: 7BWI).

## Abbreviations

hERG: human *ether-à-go-go*-related gene potassium channel 1
PD: pore domain
VSD: voltage-sensing domain
cryo-EM: cryo-electron microscopy
RP-HPLC: reverse-phase high-performance liquid chromatography
NMR: nuclear magnetic resonance
HSQC: heteronuclear single quantum correlation
TEVC: two-electrode voltage clamp
WT: wild type
rEAG1: rat *ether-à-go-go* potassium channel 1
IPTG: isopropyl β-D-1-thiogalactopyranoside
Tris: tris(hydroxymethyl)aminomethane
DTT: dithiothreitol
TEV: tobacco etch virus
TFA: trifluoroacetic acid
MALDI-TOF MS: matrix-assisted laser-desorption ionization–time-of-flight mass spectroscopy
NOESY: nuclear Overhauser effect spectroscopy
COSY: correlation spectroscopy
TOCSY: total correlation spectroscopy
DSS: 4,4-dimethyl-4-silapentane-1-sulfonate
BMRB: biological magnetic resonance bank
PDB: protein data bank
DMEM: Dulbecco’s modified Eagle’s medium
FBS: fetal bovine serum
HEPES: 2-[4-(2-hydroxyethyl)-1-piperazinyl]ethanesulfonic acid
SEM: standard error of the mean
ANOVA: analysis of variance
