# Supplementary figures and images for: Mechanism of hERG inhibition by gating-modifier toxin, APETx1, deduced by functional characterization
Source: BMC Mol Cell Biol. 2021 Jan 7;22:3. doi: 10.1186/s12860-020-00337-3 (PMC7791793; doi:10.1186/s12860-020-00337-3)

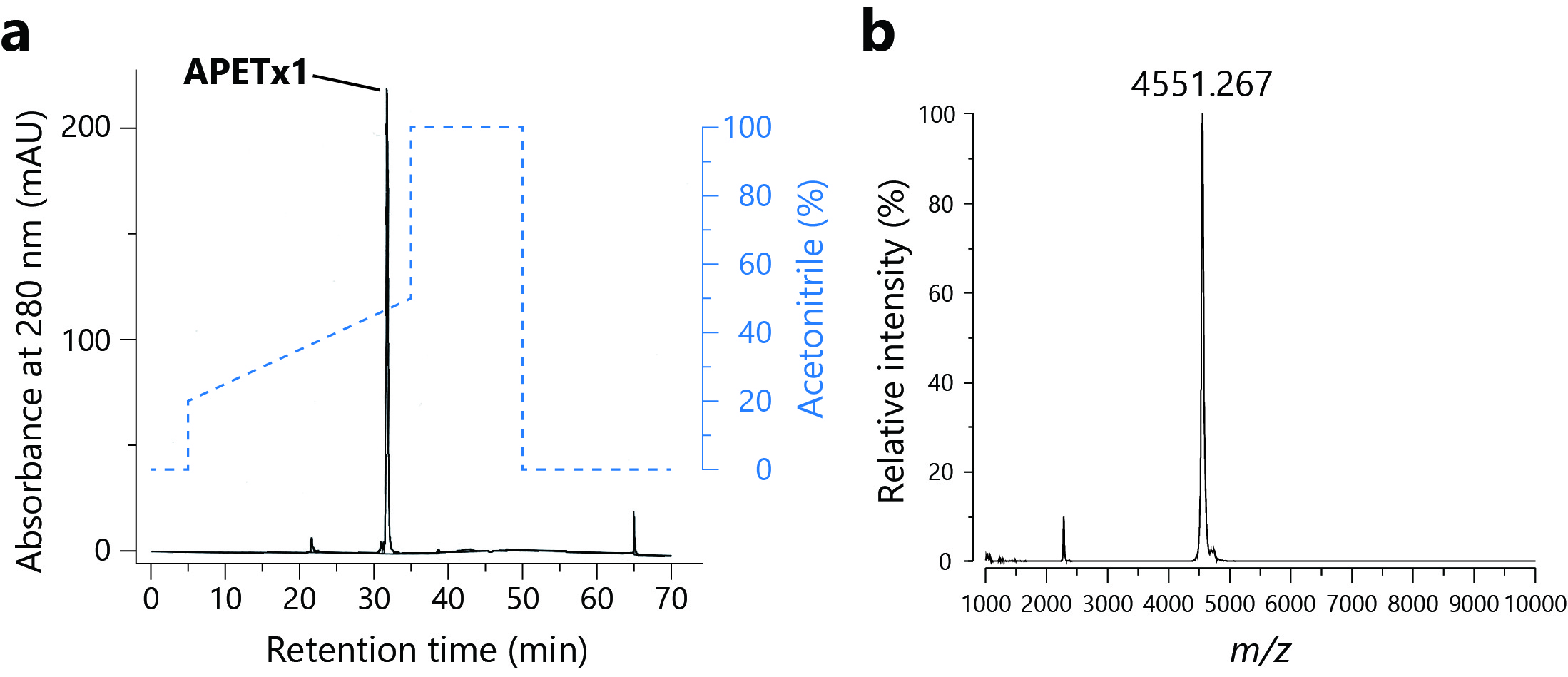

Supplement: Supplementary file 1 — Additional file 1: Figure S1. Reverse-phase HPLC and MALDI-TOF MS analysis of recombinant APETx1. (a) Chromatogram (left y-axis, black solid line) and gradient protocol (right y-axis, blue dashed line). (b) MALDI-TOF mass spectrum. [file 12860_2020_337_MOESM1_ESM.jpg]

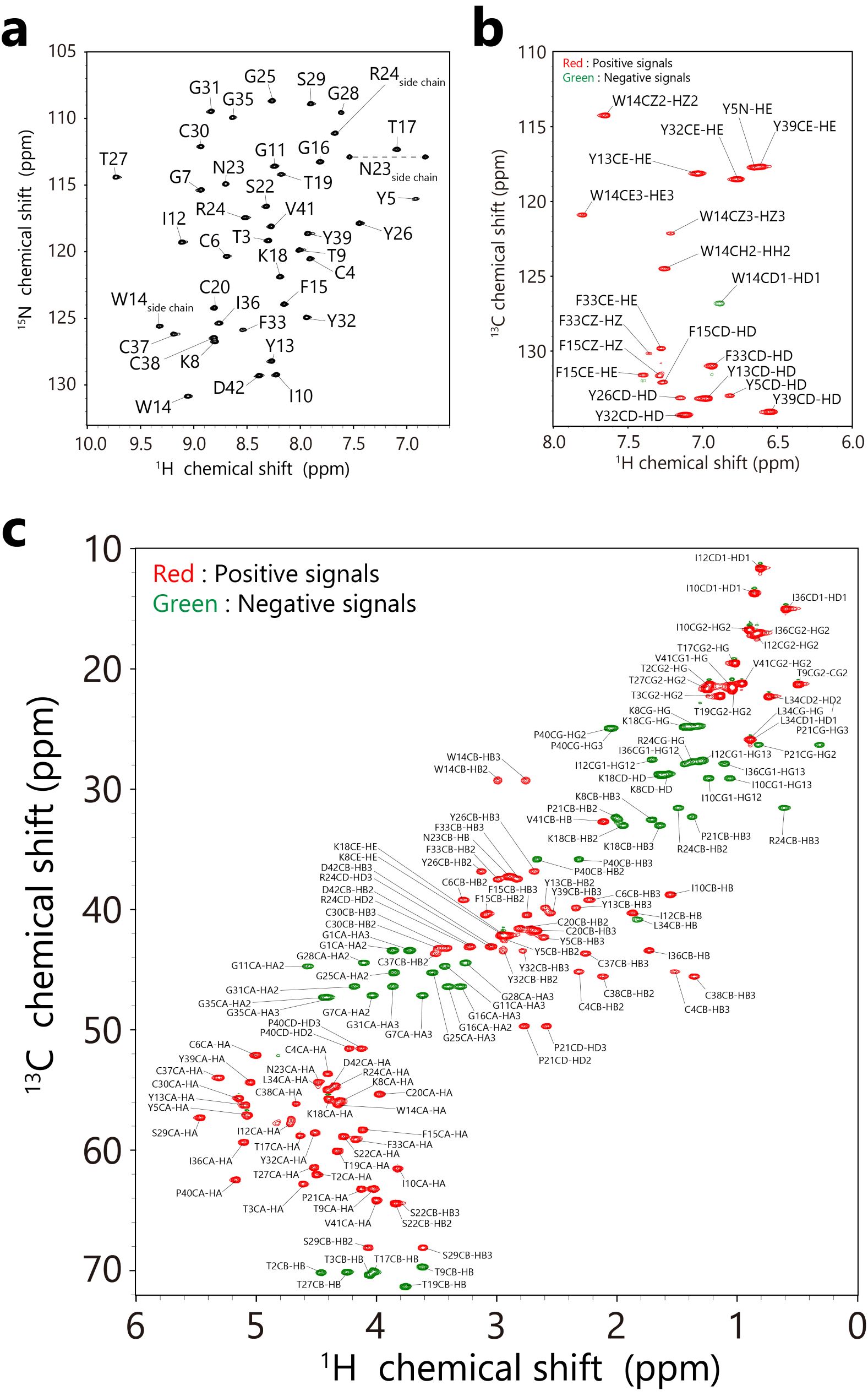

Supplement: Supplementary file 2 — Additional file 2: Figure S2. NMR resonance assignments of APETx1 on the 1H-15N HSQC and 1H-13C HSQC spectra observed at pH 6.0 and 298 K. 1H-15N HSQC spectrum (a), 1H-13C HSQC spectra for the aromatic region (b), and for the aliphatic region (c). [file 12860_2020_337_MOESM2_ESM.jpg]

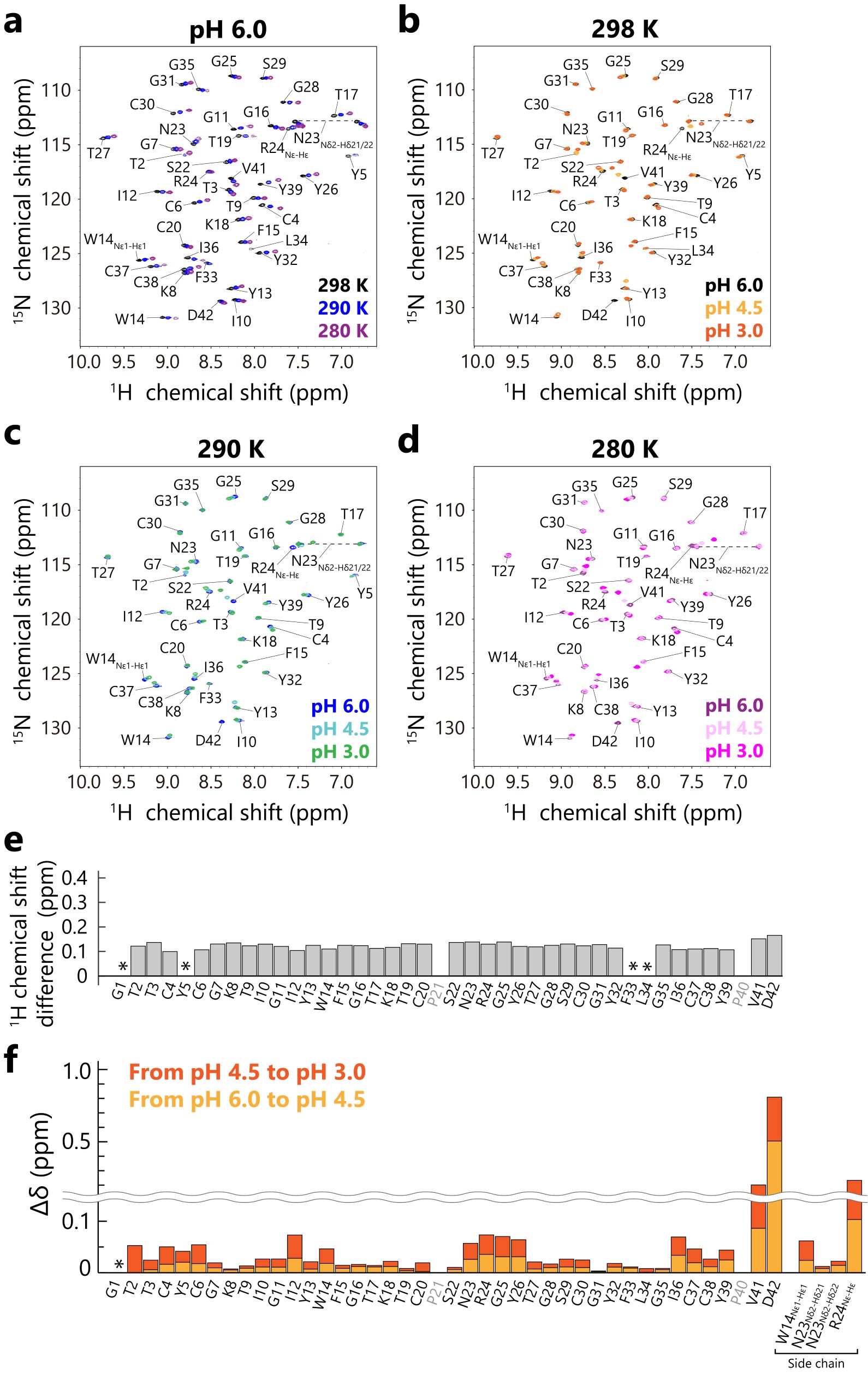

Supplement: Supplementary file 3 — Additional file 3: Figure S3. Variable-temperature and pH-titration NMR measurements using 1H-15N HSQC. (a) Overlay of the 1H-15N HSQC spectra at pH 6.0 under the different temperature conditions. (b-d) Overlay of the 1H-15N HSQC spectra at 298 K, 290 K, and 280 K in (b), (c), and (d), respectively, under different pH conditions. (e) 1H chemical shift differences of the backbone amide protons between recombinant APETx1 from the present study (BMRB ID: 36345) and the natural product from a previous study (BMRB ID: 6370) [29] under the same conditions (pH 3.0 and 280 K). The 1H chemical shift values found in the previous study were subtracted from those found in the present study. The proline residues, which lack amide protons, are labeled in gray. Asterisks (*) show that the amide signal was not observed at pH 3.0 and 280 K. (f) pH-dependent chemical shift change (Δδ) values of the 1H-15N HSQC spectral signals at 298 K (b). The Δδ values were calculated using the following equation [63]: Δδ = [(Δδ1H)2 + (Δδ15N / 6.5)2]1/2 where Δδ1H and Δδ15N are the chemical shift changes in the 1H and 15N dimensions, respectively. The proline residues, which lack amide protons, are labeled in gray. An asterisk (*) represents G1, which was not observed in the 1H-15N HSQC spectra. [file 12860_2020_337_MOESM3_ESM.jpg]

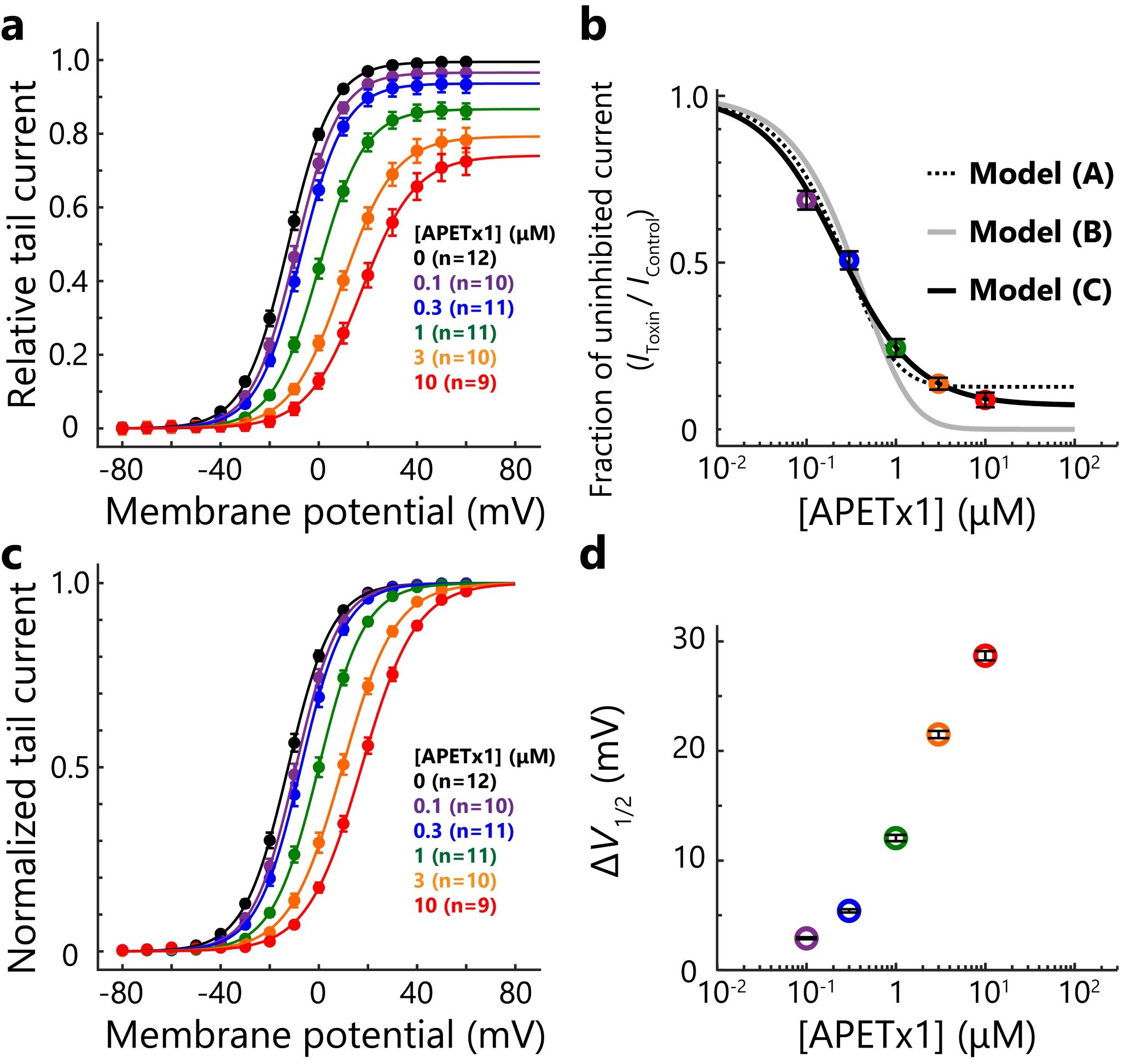

Supplement: Supplementary file 4 — Additional file 4: Figure S4. Dose-dependent effects of APETx1 on hERG in TEVC recordings. (a) G-V curves (mean ± SEM) of hERG in the presence or absence of different concentrations of APETx1. (b) Dose-response curve using the fraction of uninhibited currents at the depolarizing pulse, − 30 mV . We fitted the data with the following three models [28]: (A) four equivalent and independent binding sites per channel with fractional toxin-sensitive current, IToxin / Icontrol = Amax [Kd / (Kd + [APETx1])]4 + (1 − Amax), Kd = 1.2 μM, Amax = 0.87; (B) Four equivalent and independent binding sites per channel with fully toxin-sensitive current, IToxin / Icontrol = [Kd / (Kd + [APETx1])]4, Kd = 1.7 μM; (C) One binding site per channel with fractional toxin-sensitive current, IToxin / Icontrol = Amax [Kd / (Kd + [APETx1])] + (1 − Amax), Kd = 0.23 μM, Amax = 0.93. (c) Normalized G-V curves (mean ± SEM). (d) The ΔV1/2 values of different concentrations of APETx1. Data points and error bars represent the mean values ± SEM. [file 12860_2020_337_MOESM4_ESM.jpg]

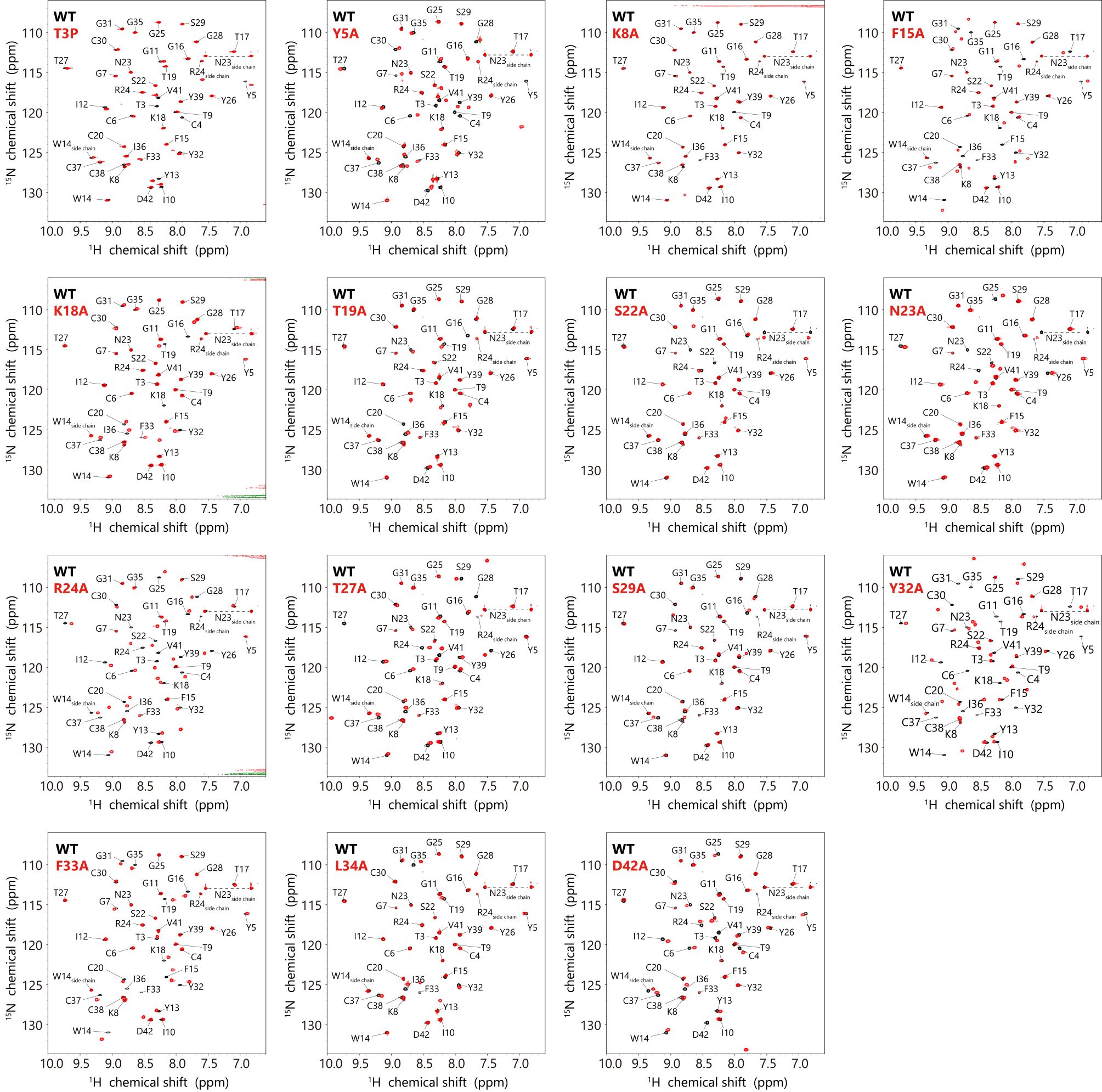

Supplement: Supplementary file 5 — Additional file 5: Figure S5. 1H-15N HSQC spectra of APETx1 mutants. All 1H-15N HSQC spectra were measured at pH 6.0 and 298 K in the following solution: T3P, K8A, F15A, K18A, R24A, Y32A, or F33A mutants, 20 mM potassium phosphate (pH 6.0), 100 mM KCl, and 10% D2O; Y5A, T19A, S22A, N23A, T27A, S29A, L34A, or D42A mutants, 10% D2O (pH 3.0); and WT recorded in both conditions. The spectrum of each mutant is superimposed onto that of WT under identical solution conditions. NMR resonance assignments were labeled according to the signals of WT. [file 12860_2020_337_MOESM5_ESM.jpg]

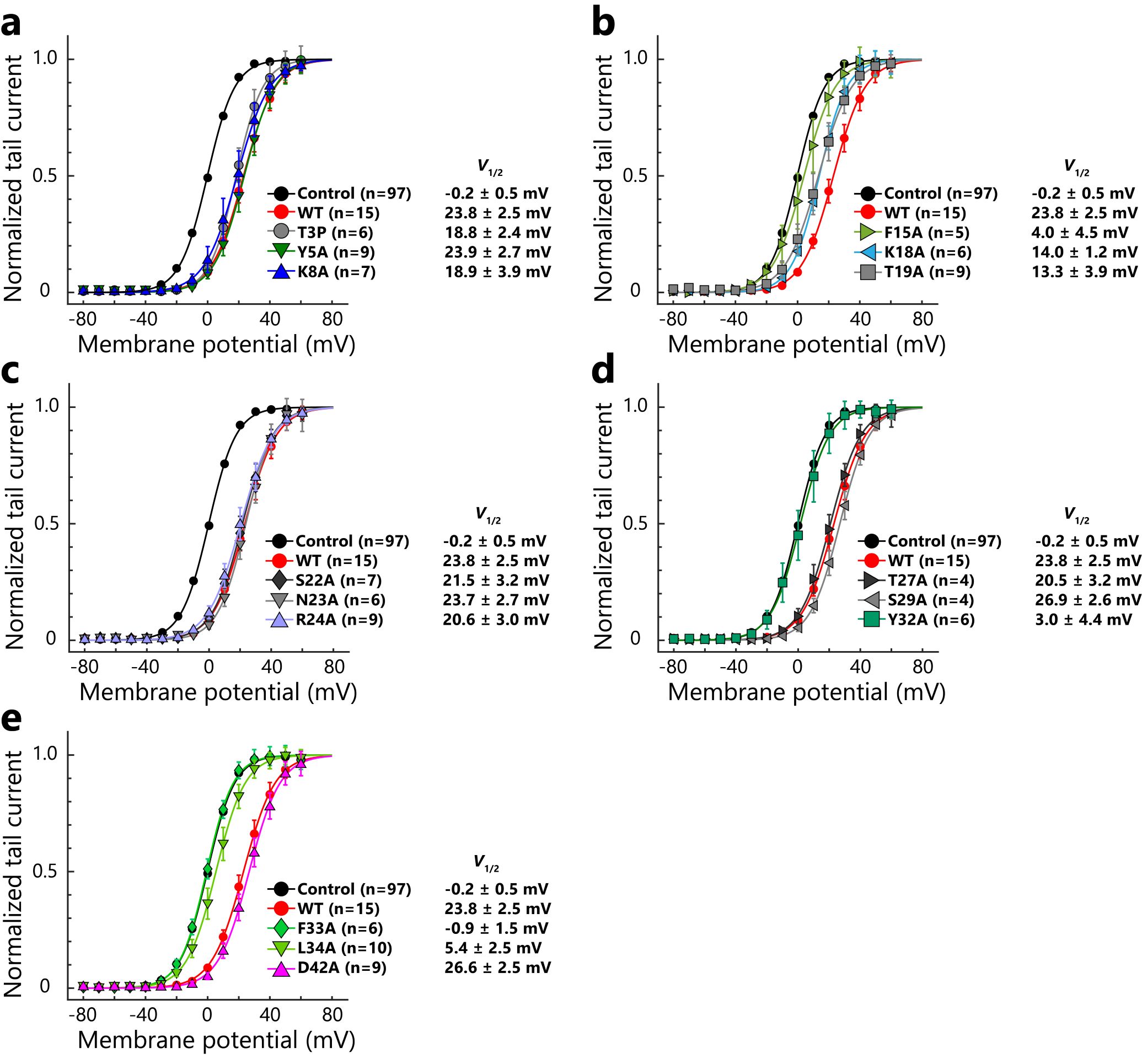

Supplement: Supplementary file 6 — Additional file 6: Figure S6. G-V curves of hERG in the presence or absence of 10 μM APETx1 and mutants. Normalized G-V curves (mean ± SEM) of hERG in the presence of 15 APETx1 mutants are sorted by amino acid residue order, divided by three into the five panels. [file 12860_2020_337_MOESM6_ESM.jpg]

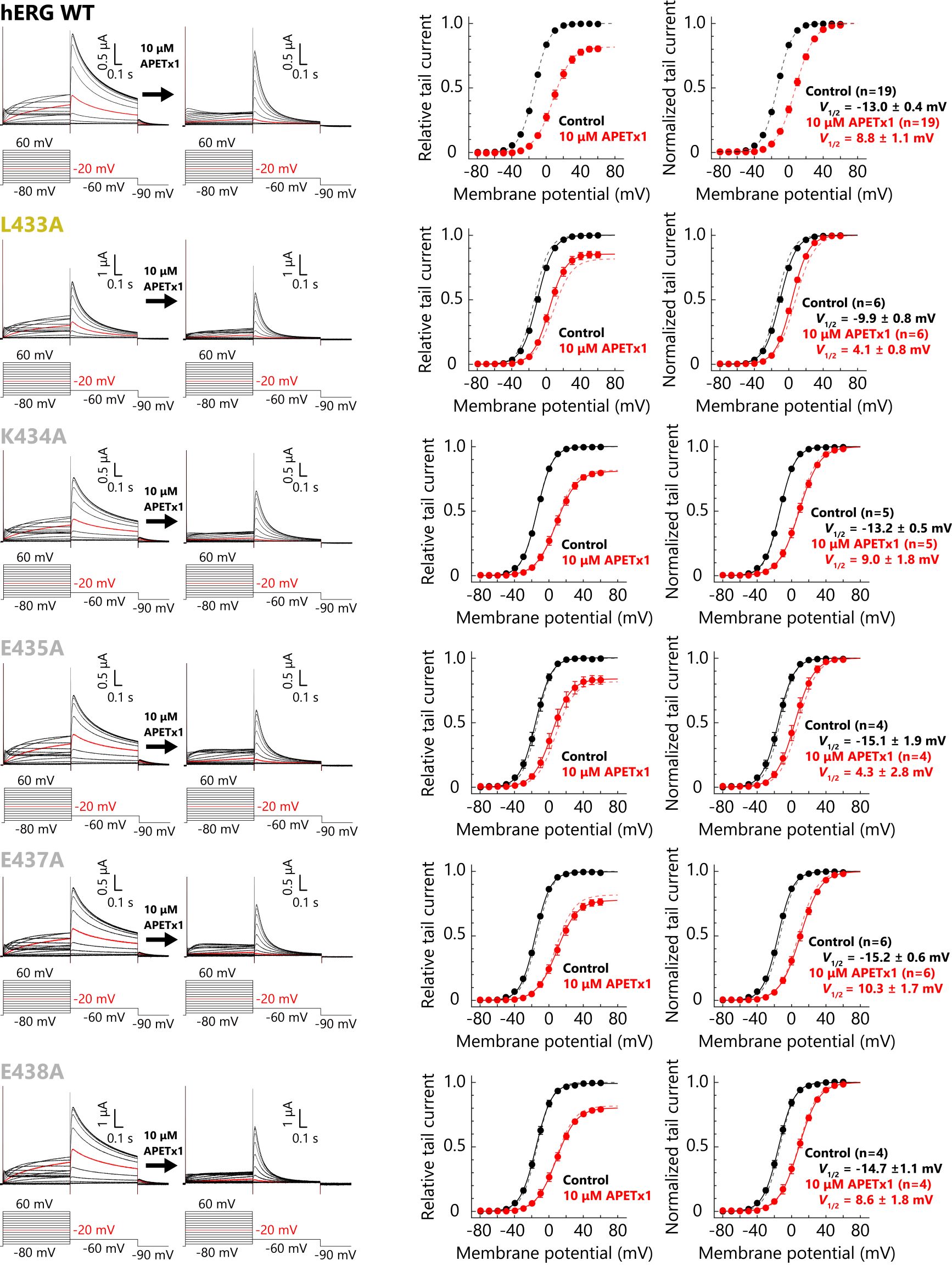

Supplement: Supplementary file 7 — Additional file 7: Figure S7. The current traces and G-V curves of hERG and its mutants in the presence or absence of 10 μM APETx1. Current traces of the hERG mutants before and after the administration of 10 μM APETx1 (left). Voltage protocol is illustrated at the bottom of each current trace. Current traces and voltage protocols at arbitrary potentials are depicted with red to clearly indicate the current reduction by the inhibitory effect of APETx1. G-V curves (mean ± SEM) of hERG mutants in the absence (black filled circle and solid line) or presence (red filled circle and solid line) of 10 μM APETx1 (right). The fitting curves of the WT in the absence (black dashed line) and presence (red dashed line) of 10 μM APETx1 are superimposed onto those of the mutants. [file 12860_2020_337_MOESM7_ESM.jpg]

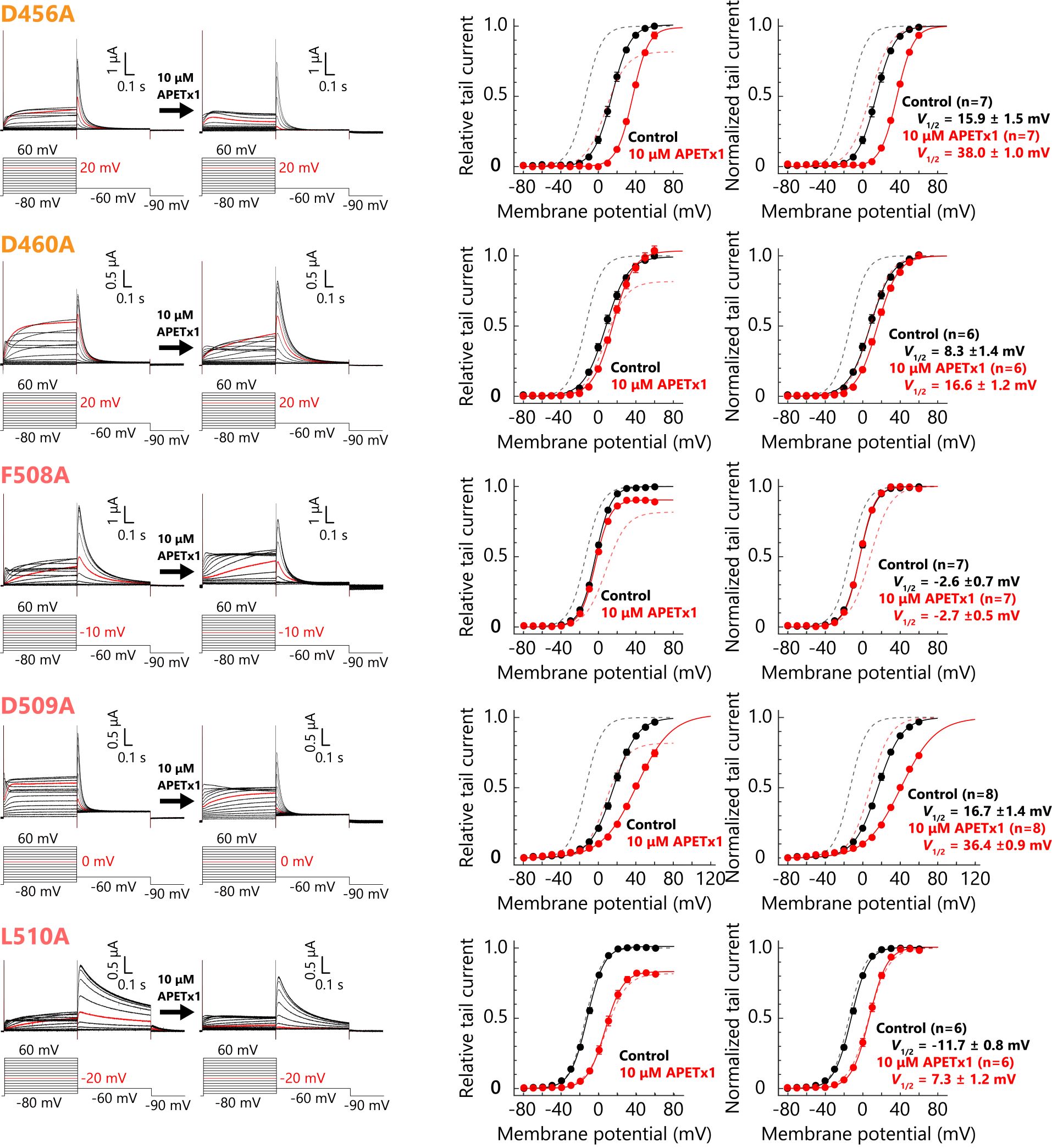

Supplement: Supplementary file 8 — Additional file 8: Figure S8. The current traces and G-V curves of hERG mutants in the presence or absence of 10 μM APETx1. Current traces of the hERG mutants before and after the administration of 10 μM APETx1 (left). Voltage protocol is illustrated at the bottom of each current trace. Current traces and voltage protocols at arbitrary potentials are depicted with red to clearly indicate the current reduction by the inhibitory effect of APETx1. G-V curves (mean ± SEM) of hERG mutants in the absence (black filled circle and solid line) or presence (red filled circle and solid line) of 10 μM APETx1 (right). The fitting curves of the WT in the absence (black dashed line) and presence (red dashed line) of 10 μM APETx1 are superimposed onto those of the mutants. [file 12860_2020_337_MOESM8_ESM.jpg]

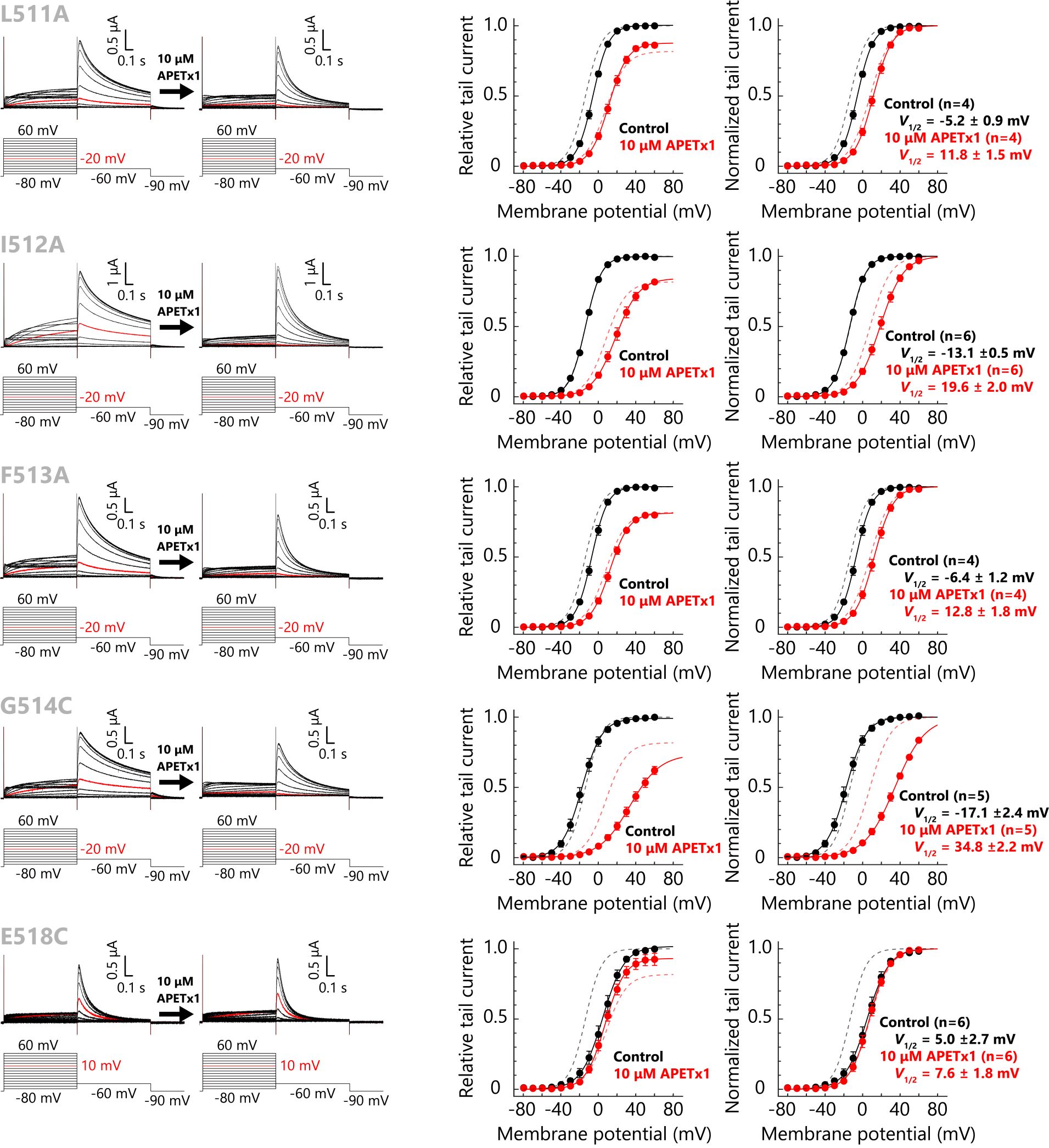

Supplement: Supplementary file 9 — Additional file 9: Figure S9. The current traces and G-V curves of hERG mutants in the presence or absence of 10 μM APETx1. Current traces of the hERG mutants before and after the administration of 10 μM APETx1 (left). Voltage protocol is illustrated at the bottom of each current trace. Current traces and voltage protocols at arbitrary potentials are depicted with red to clearly indicate the current reduction by the inhibitory effect of APETx1. G-V curves (mean ± SEM) of hERG mutants in the absence (black filled circle and solid line) or presence (red filled circle and solid line) of 10 μM APETx1 (right). The fitting curves of the WT in the absence (black dashed line) and presence (red dashed line) of 10 μM APETx1 are superimposed onto those of the mutants. [file 12860_2020_337_MOESM9_ESM.jpg]

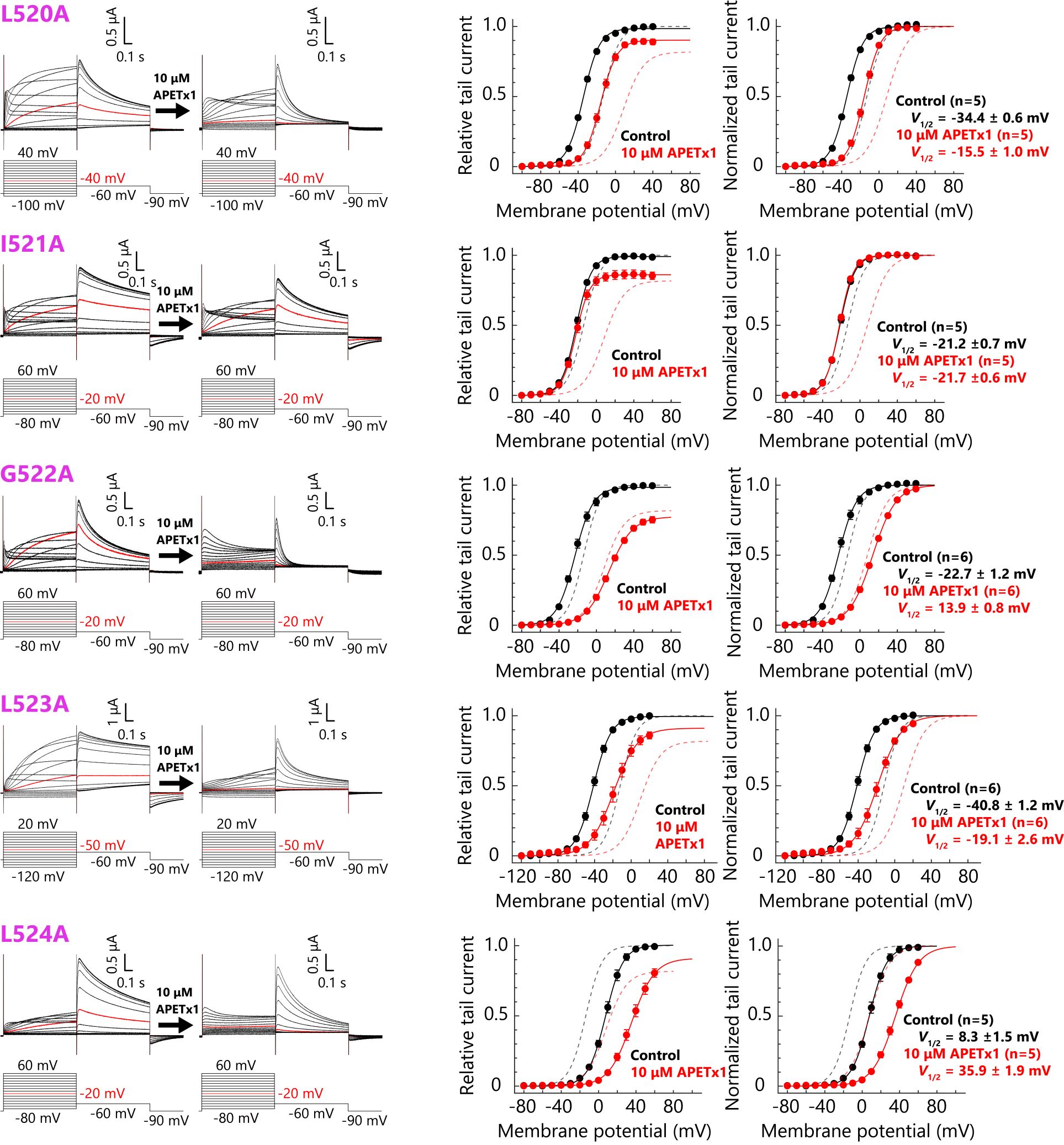

Supplement: Supplementary file 10 — Additional file 10: Figure S10. The current traces and G-V curves of hERG mutants in the presence or absence of 10 μM APETx1. Current traces of the hERG mutants before and after the administration of 10 μM APETx1 (left). Voltage protocol is illustrated at the bottom of each current trace. Current traces and voltage protocols at arbitrary potentials are depicted with red to clearly indicate the current reduction by the inhibitory effect of APETx1. G-V curves (mean ± SEM) of hERG mutants in the absence (black filled circle and solid line) or presence (red filled circle and solid line) of 10 μM APETx1 (right). The fitting curves of the WT in the absence (black dashed line) and presence (red dashed line) of 10 μM APETx1 are superimposed onto those of the mutants. [file 12860_2020_337_MOESM10_ESM.jpg]

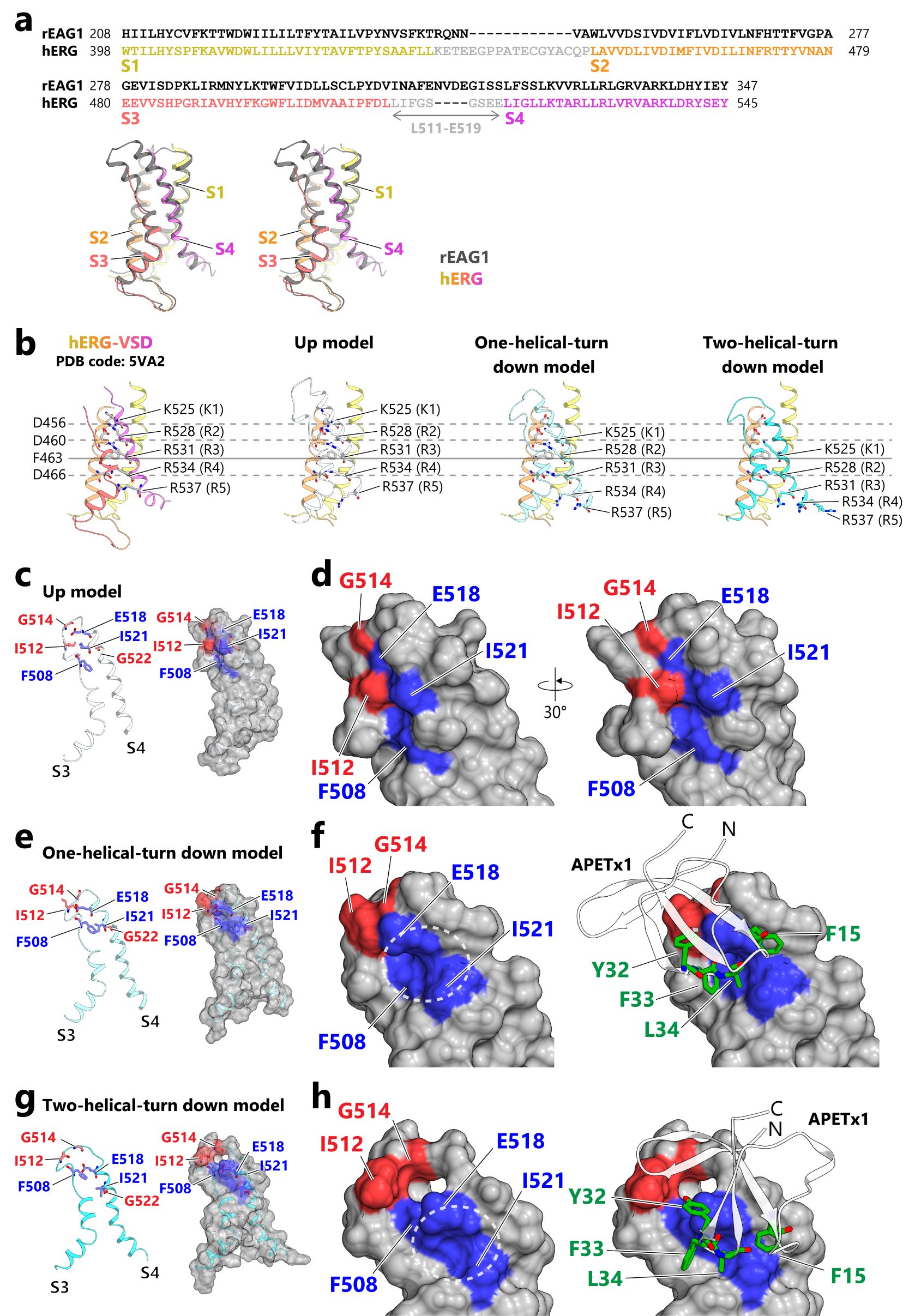

Supplement: Supplementary file 11 — Additional file 11: Figure S11. Model building of the APETx1-VSD complex. (a) Sequence alignment and stereo view of structure comparison between the VSD of hERG (The Universal Protein Resource Knowledgebase (UniProtKB) Entry: Q12809, PDB code: 5VA2) and that of rEAG1 (UniProtKB Entry: Q63472, PDB code: 5K7L). hERG is colored as in Fig. 4a, and rEAG1 appears dark gray. (b) Validation of the homology models of the hERG S3-S4 region. Ribbon representation of the cryo-EM structure of hERG (PDB code: 5VA2) [17], up model, one- and two-helical-turn down models (from left to right). Basic residues at positions K1-R5 on S4, and the gating charge transfer center residue (F463) and the acidic residues (D456, D460, and D466) on S2 are represented as sticks. To clearly show the position of S4, the Cα positions of the S2 residues are depicted with horizontal gray dashed (D456, D460, and D466) or solid lines (F463). (c) Ribbon and semi-transparent surface representations of the up model of the S3-S4 region, colored as in Fig. 5. (d) Close-up view of (c). The one- (e) and two-helical-turn down models (g) of the S3-S4 region are represented as ribbons and semi-transparent surfaces colored as in Fig. 5. On the left of (f) and (h) are close-up views of (e) and (g), respectively. White dotted circles indicate the crevice between F508, E518, and I521. On the right, docked APETx1 is also displayed as semi-transparent ribbons with the green sticks indicating key residues involved in hERG inhibition. [file 12860_2020_337_MOESM11_ESM.jpg]

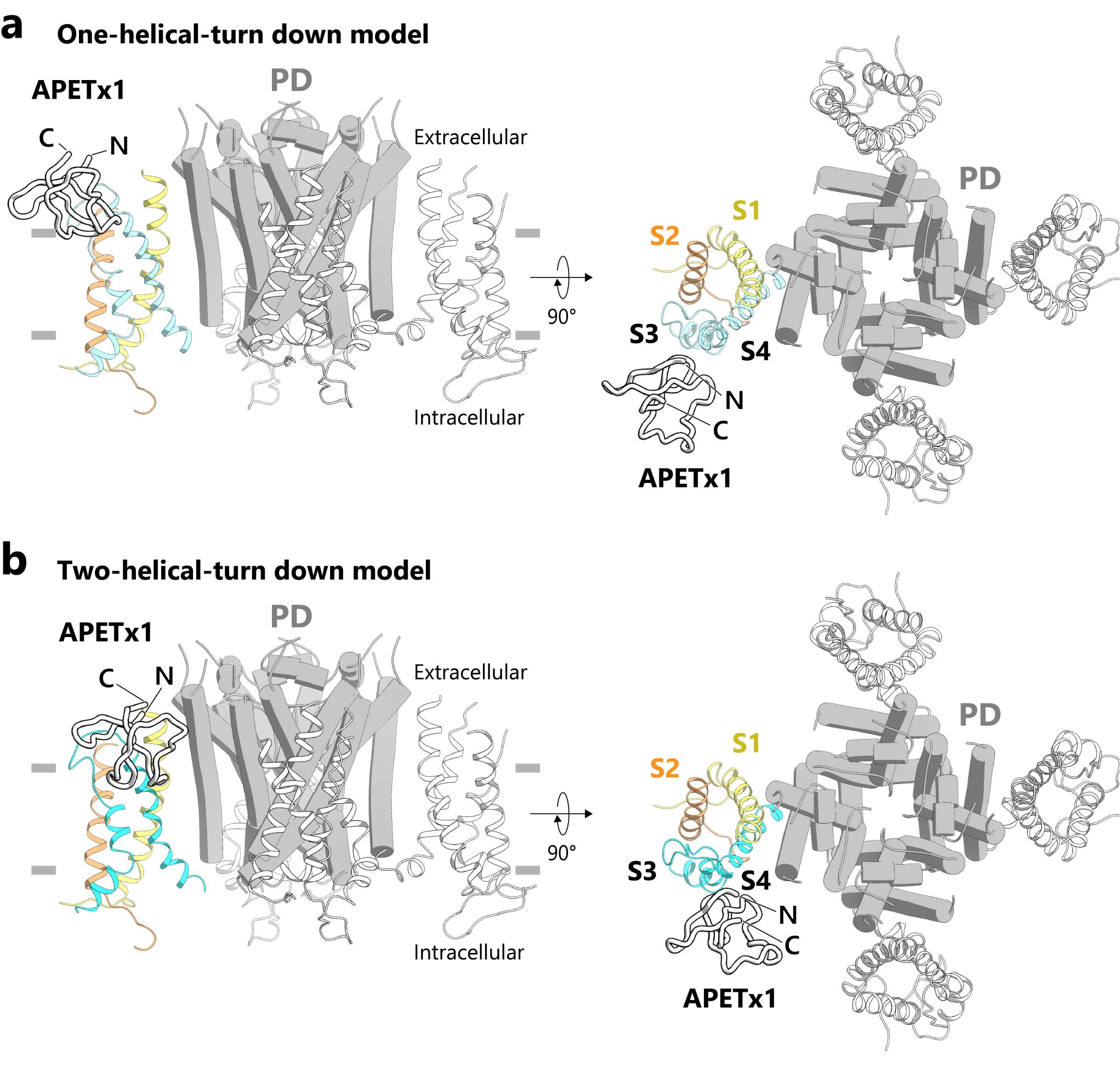

Supplement: Supplementary file 12 — Additional file 12: Figure S12. The binding location of APETx1 in a tetrameric transmembrane architecture of hERG. APETx1-VSD complex models, in which S4 adopts one- and two-helical-turn down conformations in (a) and (b), respectively, are superimposed onto the transmembrane domain of the cryo-EM of hERG (PDB code: 5VA2) [17], viewed from within the membrane plane (left) and from the extracellular side (right). One of the four VSDs of the cryo-EM structure is substituted by the APETx1-VSD complex model. The PD is represented as a cartoon, the VSDs as ribbons, and APETx1 as tubes. In the APETx1-docked VSD, S1 is yellow; S2, orange; S3 and S4 in (a), sky blue; and S3 and S4 in (b), cyan. [file 12860_2020_337_MOESM12_ESM.jpg]
